# Dental plaque-inspired versatile nanosystem for caries prevention and tooth restoration

**DOI:** 10.1016/j.bioactmat.2022.06.010

**Published:** 2022-06-21

**Authors:** Yue Xu, Yuan You, Luyao Yi, Xiaoyi Wu, Yaning Zhao, Jian Yu, He Liu, Ya Shen, Jingmei Guo, Cui Huang

**Affiliations:** a The State Key Laboratory Breeding Base of Basic Science of Stomatology (Hubei-MOST) & Key Laboratory for Oral Biomedical Ministry of Education, School & Hospital of Stomatology, Wuhan University, Wuhan, China; b Division of Endodontics, Faculty of Dentistry, The University of British Columbia, Canada

**Keywords:** Biofilms, Dental caries, Micelles, Peptide, Stimuli responsive polymers, Tooth remineralization

## Abstract

Dental caries is one of the most prevalent human diseases resulting from tooth demineralization caused by acid production of bacteria plaque. It remains challenges for current practice to specifically identify, intervene and interrupt the development of caries while restoring defects. In this study, inspired by natural dental plaque, a stimuli-responsive multidrug delivery system (PMs@NaF-SAP) has been developed to prevent tooth decay and promote enamel restoration. Classic spherical core-shell structures of micelles dual-loaded with antibacterial and restorative agents are self-assembled into bacteria-responsive multidrug delivery system based on the pH-cleavable boronate ester bond, followed by conjugation with salivary-acquired peptide (SAP) to endow the nanoparticle with strong adhesion to tooth enamel. The constructed PMs@NaF-SAP specifically adheres to tooth, identifies cariogenic conditions and intelligently releases drugs at acidic pH, thereby providing antibacterial adhesion and cariogenic biofilm resistance, and restoring the microarchitecture and mechanical properties of demineralized teeth. Topical treatment with PMs@NaF-SAP effectively diminishes the onset and severity of caries without impacting oral microbiota diversity or surrounding mucosal tissues. These findings demonstrate this novel nanotherapy has potential as a promising biomedical application for caries prevention and tooth defect restoration while resisting biofilm-associated diseases in a controlled manner activated by pathological bacteria.

## Highlights

• Nanomaterials can adhere to tooth and target acidic biofilms specifically.
• Application of caries prevention and tooth defect restoration.
• Guidance for the innovation of the existing post-defect restoration strategies.
• The multidrug delivery system exerts antibacterial and restorative abilities on demand.
• Bacteria-responsive system resists biofilm-associated diseases in a controlled manner.

## Graphical abstract

**Figure:**
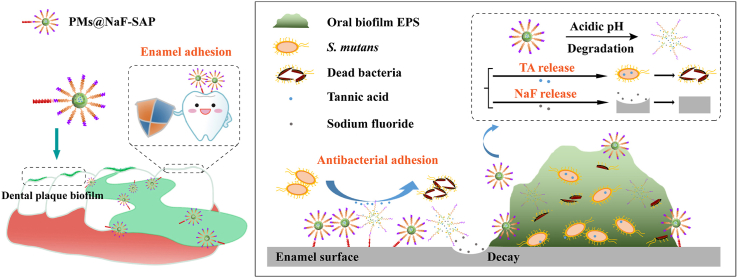

## 1 Introduction

Dental caries is currently one of the most common and prevalent human diseases affecting over 3.5 billion people globally [[1], [2], [3]], where 60–90% of children and the majority of adults are affected worldwide, thereby yielding considerable financial and quality-of-life burdens [[4], [5], [6]]. In particular, the effects of tooth decay are not restricted to oral symptoms. Studies have shown that children with severe early childhood caries have poorer nutritional health compared to children without caries [6,7]; infection and sepsis due to the spread of caries to the pulp can sporadically lead to severe systemic consequences, such as the spread of localized infections or even treatment-related deaths (e.g., complications of anesthesia), which poses a major public health challenge [8]. Dental caries is a multifactorial, plaque-evoked, dynamic disease that results in the destruction of dental hard tissues [9]. Salivary proteins adhere to the tooth surface and trigger the initial plaque establishment, forming the pellicle a substrate for bacterial attachment. The demineralization caused by the acid production of bacteria in dental plaque biofilms thereby results in caries [10,11]. *Streptococcus mutans* (*S. mutans*), as the main cariogenic bacterium, can embed in biofilm substrates and create a strongly acidic microenvironment with a pH below 5.0, which erodes hard tooth apatite, causing the onset of tooth decay [10,12]. Traditional antimicrobials, which are primarily based on broad-spectrum antibiotics, are ineffective to a certain extent due to their limited efficacy against plaque biofilms that are well known to be difficult to eliminate or treat [9,10,[13], [14], [15]]. Additionally, due to the buffering effect of saliva in oral cavity, drugs cannot be retained for a long time to exert their efficacy. Therefore, more efficient strategies are needed to target the biological traits of the acidic plaque biofilms.

Recently, intelligent stimuli-responsive nanomaterials have provided remarkable specificity and versatility as efficient drug delivery vehicles for biomedical applications, including antibacterial adhesion [16,17] and biofilm disruption [18,19], which can achieve on-demand release to improve bacterial targeting and local drug concentration, thereby reducing side effects and bacterial resistance [20,21]. In particular, bacteria-responsive systems offer the possibility to respond to dental caries, where the acidic biofilm microenvironment caused by cariogenic bacterial plaque acts as the trigger for controlled drug release. Thus, such intelligent nanosystems can exploit the apparent pathophysiological pH fluctuations displayed by caries in a controlled, bacteria-dependent manner, where their antimicrobial property is activated at the specific acidic pH caused by cariogenic plaque biofilms but weakened at near-neutral (physiological) pH values, improving bacterial targeting of drugs [22].

Antimicrobial and antiplaque properties are important but insufficient for caries to restore demineralized teeth [23,24]. Current restorative therapies, including restoring defects with resin, metal, and ceramics [25], are primarily post-defect restoration strategies that cannot prevent and mitigate root causes. Some new materials, such as polyacrylate acid (PAA) [26,27], casein phosphopeptide (CPP) [28,29], have been used for dental remineralization. However, these formulations are limited to short-term drug releases and cannot effectively restore defects as soon as enamel demineralization begins. Additionally, a lack of consideration of the stubborn presence of cariogenic biofilms in the demineralization lesions of real caries and the continuous bacterial challenge in the oral cavity limit the clinical application of these materials [30]. Novel nanotechnologies, along with biofilm control, are expected to promote noninvasive restorative strategies for tooth decay [31].

**Fig. 1:**
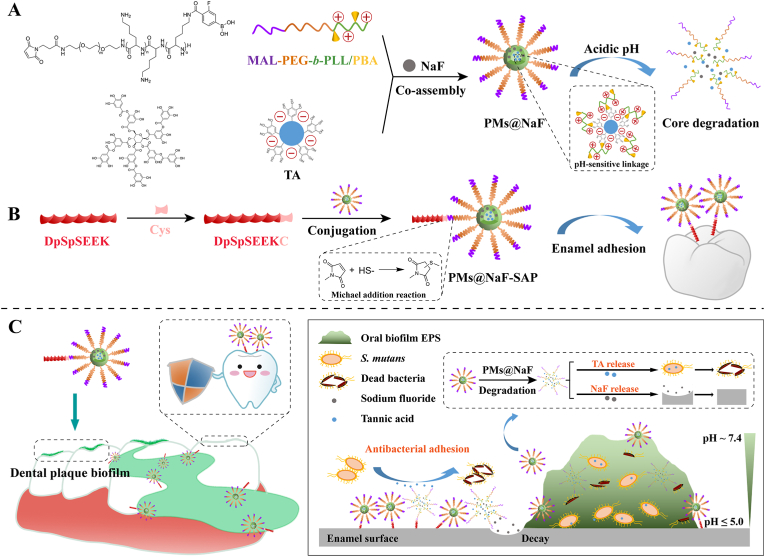
Proposed concept of the bacteria-responsive micellar multidrug delivery system (PMs@NaF-SAP) targeting cariogenic biofilm. (A) Illustration of formulation and the bacteria-responsive activity of PMs@NaF. (B) The modification of SAP and the formulation of PMs@NaF-SAP. (C) Schematic illustration of topical application of PMs@NaF-SAP on dental plaque biofilm and proposed mechanism for caries prevention and enamel restoration.

In this study, we construct a dental plaque-inspired micellar multidrug delivery system (PMs@NaF) that provides intrinsic anti-caries and restorative properties in a bacteria-triggered manner to prevent the prevalent and costly oral disease. Considering the buffering effect of saliva, salivary-acquired peptide DpSpSEEK (SAP), the vital peptide sequence from the salivary acquired pellicle [32,33], is designed to be conjugated to the constructed nanoparticles (PMs@NaF-SAP) for selective adhesion onto the tooth enamel surface (Fig. 1). The self-assembled nanoparticle system exhibits the classical nanoscale, spherical core-shell structure of micelles and can thus encapsulate multiple drugs and penetrate into the biofilm matrix. Drug release studies reveal that PMs@NaF-SAP rapidly releases antibacterial and restorative agents under acidic conditions when caries occur. The efficacy and retention time of drugs in the oral cavity can be enhanced markedly by PMs@NaF-SAP through the effective binding of SAP to tooth enamel. For its role in combatting cariogenic biofilms, we investigated its antibacterial activity, biofilm targeting, and restoration effect. The rodent cariogenic model confirms topical treatment with PMs@NaF-SAP potently inhibits biofilm formulation and acid damage of tooth enamel to efficiently prevent the occurrence and severity of caries. Thus, we propose a therapeutic platform that achieves highly efficient biofilm disruption, enamel restoration, and on-demand drug release in a pH-dependent manner induced by cariogenic bacteria without impacting the oral microflora diversity or surrounding mucosal tissues. This nanotherapy can facilitate clinical translation as a novel topical application to prevent dental caries and restore tooth defects, thereby providing a major challenge to biofilm-related diseases in a controlled manner that is activated by pathological bacteria.

## 2 Materials and methods

### 2.1 Materials

MAL-PEG-NH_2_, ε-(benzyloxycarbonyl)-l-lysine *N*-carboxyanhydride, trifluoroacetic acid (CF_3_COOH), *N*, *N*-Dimethylformamide (DMF), trichloromethane (CHCl_3_), hydrobromic acid (HBr), D-mannitol, 3-fluoro-4-carboxy-phyenylboronic acid (FPBA), 4-(4,6-dimethoxy-1,3,5-triazin-2-yl)-4-methylmorpholinium chloride n-hydrate, tannic acid (TA), sodium fluoride (NaF), chlorhexidine (CHX), and triethylamine (TEA) were purchased from Shanghai Aladdin Biochemical Technology Co., Ltd. Peptide sequence DpSpSEEKC was customized from Shanghai Top-peptide Co., Ltd. (China). *S. mutans* Ingbritt was provided by the School of Stomatology, Wuhan University (China). Brain heart infusion (BHI) broth was purchased from Beijing Land Bridge Technology Co., Ltd. (China). Mitis Salivarius Bacitricin Agar (MSBA) Base was purchased from Shandong Tuopu Biol-engineering Co., Ltd. CCK-8 assay reagent (Beijing Labgic Technology Co., Ltd) and LIVE/DEAD BacLightTM Bacterial Viability Kit L-7012 (Invitrogen) were used according to the manufacturer's instructions. All chemicals were of analytical grade.

### 2.2 Synthesis of polymer nanocarriers

#### 2.2.1 Synthesis of MAL-PEG-b-PLL/PBA

3-maleimidopropionic acid-poly(ethylene glycol)-block-poly(l-lysine)/phyenylboronic acid (MAL-PEG-*b*-PLL/PBA) was synthesized according to Fig. S1. Briefly, ε-(benzyloxycarbonyl)-l-lysine *N*-carboxyanhydride (Lys(Z)-NCA) (2.0 g) was dissolved in DMF (30 mL), and MAL-PEG-NH_2_ (2.0 g) was added and stirred under dry argon at 35 °C for 72 h, resulting in a polymerization reaction. The solvent was rotary evaporated and then dissolved in CHCl_3_, followed by placement in excess ether for precipitation. The product MAL-PEG-*b*-PZLL was obtained.

MAL-PEG-*b*-PZLL (2.0 g) was then dissolved in CF_3_COOH (20 mL) and added to HBr (2 mL). The mixture was stirred at 0 °C for 2 h and then placed in cold ether to precipitate out. This precipitate was dissolved in DMF and then reprecipitated in excess ether to remove the residual CF_3_COOH. The product MAL-PEG-*b*-PLL was dried under vacuum and obtained.

Then, MAL-PEG-*b*-PLL/PBA was prepared as follows. MAL-PEG-*b*-PLL (100 mg) was dissolved in D-mannitol (10 mL, 100 mg) in sodium bicarbonate solution (50 mM, pH 8.5), and FPBA (1.7 mL) in CH_3_OH was added. Then, 4-(4,6-dimethoxy-1,3,5-triazin-2-yl)-4-methylmorpholinium chloride n-hydrate (DMT-MM) (250 mg) was added as a coupling agent, and the mixture was stirred for 12 h to allow the conjugation of FPBA and MAL-PEG-*b*-PLL by acylation. The reaction solution was dialyzed (MWCO 3500) against NaOH solution (0.01 N), HCl solution (0.01 N) and deionized water. The product MAL-PEG-*b*-PLL/PBA was obtained by freeze-drying.

#### 2.2.2 Synthesis of PMs@NaF

PMs@NaF was synthesized by mixing MAL-PEG-*b*-PLL/PBA and TA in NaF aqueous solution. MAL-PEG-*b*-PLL/PBA (5.0 mg) was dissolved in deionized water (4 mL), and then, NaF aqueous solution (548 μl, 5 mg mL^−1^) was added. The mixture was stirred until the solute was completely dissolved. Then, TA (274 μl, 5 mg mL^−1^) in water was introduced dropwise into the above reaction mixture. After stirring at room temperature for 4 h, micelles were formed, with the solution changing from transparent to oyster white (Fig. S4). Finally, the micelle suspension was purified by centrifugation using an ultrafiltration tube (MWCO 3500), obtaining the product PMs@NaF.

#### 2.2.3 Synthesis of polymeric micelles without NaF loaded (PMs)

As a control, PMs were prepared via a technique similar to that described above. MAL-PEG-*b*-PLL/PBA (5.0 mg) was dissolved in deionized water (4 mL), followed by TA (274 μl, 5 mg mL^−1^) in water added dropwise. After stirring at room temperature for 4 h, micelles were formed. Finally, the micelle suspension was purified by centrifugation using an ultrafiltration tube (MWCO 3500), obtaining the product PMs (MAL-PEG-*b*-PLL/PBA+TA). The only difference between PMs and PMs@NaF is that no NaF aqueous solution was added (i.e., a micellar particle structure with only TA loaded).

#### 2.2.4 Conjugation of PMs@NaF and peptide sequences DpSpSEEKC (PMs@NaF-SAP)

The peptide sequence DpSpSEEK was first modified with a cysteine at the end, thereby forming DpSpSEEKC, which could be conjugated to PMs@NaF through a maleimide-sulfhydryl reaction. Briefly, DpSpSEEKC (1.0 mg) was added to the solution of the above purified PMs@NaF in TEA (pH 8.0). Then, the solution was stirred vigorously for 2 h at room temperature. After the reaction, the system was purified by centrifugation using ultrafiltration tubes (MWCO 3500). Finally, the product PMs@NaF-SAP was stored at 4 °C before use.

### 2.3 Characterization of polymers

The morphologies of MAL-PEG-*b*-PLL/PBA, PMs@NaF and PMs@NaF-SAP were observed using transmission electron micrographs (TEM, JEM-100SX, Japan). The particle size and zeta potential of polymers were measured using dynamic light scattering (DLS, Zetasizer Nano ZS90, Malvern Instruments, Malvern, UK). The concentrations of each polymer were measured by lyophilization. The molecular weight and polydispersity index (PDI) of the samples were determined by Gel Permeation Chromatography (GPC, Agilent PL-GPC50/Agilent 1260). The FT-IR spectra of the samples were recorded using a fourier transform infrared spectrometer (FTIR spectrometer, NICOLET 5700) to confirm the successful conjugation between PMs@NaF and SAP. After dissolving in DMSO-d_6_, the ^1^H NMR spectra of MAL-PEG-*b*-PLL/PBA and PMs were recorded using a Bruker Avance spectrometer (Bruker AV II-400 MHz).

### 2.4 In vitro drug loading and release

#### 2.4.1 Drug loading

The drug loading content (DLC) and drug loading efficiencies (DLE) of PMs@NaF and PMs@NaF-SAP were calculated using ion exchange chromatography (IEC, ICS2500, DIONEXFEI, USA) and high-performance liquid chromatography (HPLC, Thermo Fisher Scientific U3000). The micellar solution was dissolved in ethanol for demulsification to allow the complete release of drugs from PMs with a volume ratio of 1:4. The TA concentration was quantified by HPLC, and the detection wavelength was 276 nm. The calibration curve was preestablished to calculate the content of TA. Similarly, IEC measurements allow the concentration of NaF to be monitored. DLC and DLE were calculated using the equation:

(1) Drug loading content (%) = (Weight of drug in micelles)/(Total weight of micelles) × 100

(2) Drug loading efficiency (%) = (Weight of drug in micelles)/(Weight of the feeding drug) × 100

#### 2.4.2 In vitro pH-responsive drug release

The drug release of PMs@NaF and PMs@NaF-SAP was investigated using the dialysis method. The changes in TA release from PMs could be detected by HPLC under different pH conditions due to boronic acid-catechol interactions. PMs@NaF or PMs@NaF-SAP (2 mL) were transferred into a dialysis bag (MWCO 3500) and subsequently immersed into 15 mL of PBS at pH values of 5.0 and 7.4 with gentle shaking (120 rpm, 37 °C). At predetermined time points (0.5, 1, 2, 4, 8, 12 and 24 h), the release medium (2 mL) was removed for HPLC measurement and replaced with an identical volume of fresh medium. The release activities were measured at the characteristic absorbance of 276 nm according to the preestablished calibration curve of TA.

Similarly, due to the dissociation of micelles in an acidic environment, NaF could also be released from PMs. The NaF release of PMs@NaF and PMs@NaF-SAP was measured under three different pH conditions (pH 5.0, 6.5 and 7.4). The monitoring procedure was prepared via the same method as above. The concentration of NaF was quantified by IEC. Each experiment was performed in triplicate, and the results are presented as the mean ± SD.

### 2.5 Adsorbability of PMs@NaF-SAP on human enamel samples

Freshly extracted, non-carious human third molars were collected after receiving approval from the Ethics Committee of the School and Hospital of Stomatology, Wuhan University. The teeth were stored in 0.1% thymol at 4 °C before specimen preparation. The crowns were sectioned with a low-speed, water-cooled diamond saw after separation from the roots. Then, all crowns were separated longitudinally to obtain buccal and lingual surfaces. Enamel surfaces as the working side were ground flat and then polished with SiC emery paper under running deionized water, with the other sides painted with acid-proof nail polish. After polishing, these specimens were ultrasonically cleaned in deionized water for 15 min to remove any residue. The samples were stored at 4 °C in deionized water before use.

To explore the adsorption capability of PMs@NaF-SAP on human enamel, we used 5-carboxyfluorescein (Fam)-labeled SAP to visualize the binding and localization of nanoparticles on the enamel surface. One milliliter of PMs@NaF, Fam-labeled SAP, and PMs@NaF-SAP solution was added dropwise to the human enamel sample surface and kept for 30 min with rocking in the dark at 37 °C. Then, the samples were rinsed three times with PBS to remove unbound materials and dried in a vacuum oven. The adsorbed enamel samples were observed by confocal laser scanning microscope (CLSM, ZEISSLSM700, Germany).

The adsorption time of PMs@NaF-SAP on human enamel was also tested using CLSM. Enamel working surfaces were exposed to Fam-labeled PMs@NaF-SAP solution (1 mL) for 30 min, followed by washing with PBS 3 times as described above. Then, the samples were transferred into equivalent PBS solutions and were placed onto a rotator at 37 °C in the dark. After incubation for 0, 0.5, 1, 2, 3, 5, and 7 d, the samples were removed and freeze-dried separately. Finally, they were also measured by CLSM.

### 2.6 In vitro antibacterial and antibiofilm experiments

#### 2.6.1 Antibacterial activity

*S. mutans* UA159, as a virulent cariogenic pathogen and well-characterized biofilm-forming strain, grew to the mid-exponential stage in BHI at 37 °C and 5% CO_2_. Then, actively growing *S. mutans* suspension (40 μl, 10^9^ CFU ml^−1^) along with PMs@NaF-SAP (160 μl in PBS at pH 5.0, 6.5 and 7.4) were pipetted into 96-well cell culture plates [200 μl well^−1^]. After incubation at 37 °C for predetermined times (0.5, 1, 2, 4, 8, 12, and 24 h), 100 μl of cell suspension from each well was transferred to a new 96-well plate, and the absorbance was measured at 600 nm via a microplate reader to detect cell growth. CHX and PBS with bacterial cells served as the positive control and negative control, respectively. All measurements were conducted in triplicate experiments, and each timing was tested in three wells.

#### 2.6.2 Biofilm inhibition assay

The effects of PMs@NaF-SAP on developing *S. mutans* biofilm formation were assessed by a similar treatment regimen as above. An overnight culture of *S. mutans* was subcultured at an OD_600_ of 0.5 into BHI containing 1% (w/v) sucrose (BHIS) medium in a 96-well plate to form the biofilm. PMs@NaF-SAP (100 μl) in BHIS at pH 5.0, 6.5 or 7.4 was then added to the wells. After incubation at 37 °C for predetermined times (0.5, 1, 2, 4, 8, 12, and 24 h), any excess medium in each well was discarded, and the plates were gently washed with sterile PBS to remove any planktonic bacterial cells. Biofilm inhibition was determined by crystal violet staining. Methanol (100 μl, 10%) was used to fix bacterial cells for 30 s in each well. Then, the methanol was removed, and crystal violet dye (100 μl) was added at room temperature. After 20 min, the wells were rinsed at least four times with sterile water to remove unbound dye, followed by intensive drying at 37 °C. Then, 33% (w/v) acetic acid (100 μl) was added to each well to dissolve the dye, and the cells were incubated in the dark while undergoing shaking for 15 min to release the dye. Finally, absorbance was measured at 595 nm to assess the differences among treated samples for each tested pH or time. The relative capacity of biofilm (%) was defined as the degree of reduction in biofilm formation compared to untreated cells. CHX was used as a positive control, and BHIS with cells was used as a negative control, respectively. Setting the negative control group as 100% biofilm growth, the other groups/negative group refers to the relative capacity of biofilm (%). All experiments were performed in triplicate, and each timing was tested in three wells.

#### 2.6.3 Contact angle measurement

Hydroxyapatite discs (HA discs, 5 mm in diameter, 2 mm in thickness) were manufactured using a tablet press (HY-12, Tianjin Tianguang Optical Instrument Co., Ltd.) and were under high-pressure steam sterilization prior to tests. Then, the discs were coated with PMs@NaF-SAP or ddH_2_O for 30 min, followed by vacuum drying at room temperature. A goniometer (JY-82B Kruss DSA, Dataphysics OCA20, China) was used to measure the contact angle between deionized water and the surface of the multilayer coating. On each sample, there were water droplets for imaging and were analyzed by a photocoupled component (CCD) camera (IMT 3, IMT Solutions), ImageJ software and LB-ADSA plug-in for contact angle analysis. The contact angle calculation was based on three measurements on each disc, and the average value of each group of 3 samples was taken.

#### 2.6.4 Antibacterial adhesion ability

First, HA discs were sterilized and divided into two groups: one was coated with filter-sterilized human entire saliva at 37 °C for 1 h (sHA), and the other was left untreated (HA). Then, the HA and sHA discs were immersed in PMs@NaF-SAP, PMs@NaF, SAP solutions or sterile deionized water (control) (400 μl) for 30 min at 37 °C. After each treatment, the discs were washed with sterile PBS 3 times to remove excess and unbound materials. They were then placed in a 48-well plate, followed by the addition of *S. mutans* in BHI (400 μl, 10^6^ CFU mL^−1^). After incubation at 37 °C for 1.5 h, the HA and sHA discs were washed once with PBS. These discs were then immersed in BHIS culture medium (800 μl) for further incubation for 24 h. For the biofilm at the end of the experimental period, the HA and sHA discs were gently washed with PBS 3 times to remove unattached bacteria, and four tests were performed to analyze the antibacterial adhesion ability of PMs@NaF-SAP. First, a LIVE/DEAD BacLightTM Bacterial Viability Kit L-7012 was applied to visualize the ability of PMs@NaF-SAP to penetrate into the biofilm, with SYTO 9 (485/498 nm; Molecular Probes) and propidium iodide (PI) (535/617 nm; Molecular Probes) used for live and dead cell labeling, respectively. Fluorescence images were observed via CLSM equipped with a 40 × objective lens. Three points from each specimen were scanned at intervals from the bottom to the top of the biofilm. Images were analyzed by ImageJ. In addition, the ratio of live and dead bacterial cells was also computed based on the value of relative fluorescence intensity with Imaris 7.4.2 for quantitative analysis. In a separate experiment, the adherent biofilms were collected via sonication (30 s pulse; SB-120D, NINGBO SCIENTZ BIOTECHNOLOGY CO., LTD); the ultrasonic treatment process did not kill bacterial cells while providing optimum dispersal and maximum recoverable counts. The homogenized biofilm suspensions were serially diluted and inoculated on BHI agar plates in triplicate for CFU counting (Fig. S11). Finally, the fourth part of the biofilm experiment was for crystal violet staining (Fig. S12). Three independent experiments were conducted, and the data are presented as the mean ± SD.

#### 2.6.5 In vitro cariogenic biofilm model

Sterile HA discs were prepared as described above. First, the discs were coated with filter-sterilized human entire saliva at 37 °C for 1 h. Then, the sHA discs were placed in a 48-well plate. S. mutans was grown in BHI at 37 °C and 5% CO_2_ to mid-exponential phase, followed by dilution to 10^6^ CFU ml^−1^. Each plate was seeded with bacterial suspension (400 μl) to ensure covering the discs. After incubation at 37 °C and 5% CO_2_ for 1.5 h, which allowed the *S. mutans* to adhere to the sHA surfaces, discs were transferred to a new 48-well plate, followed by the addition of BHIS (800 μl). The cariogenic biofilm model was established until the end of the incubation period (24 h). The biofilms continued to be tested and analyzed for PMs@NaF-SAP penetration and bacterial killing as described below.

#### 2.6.6 Cariogenic biofilm resistance

Aside from antibacterial adhesion, the cariogenic biofilm resistance of PMs@NaF-SAP was assessed based on the biofilm model. The sHA discs coated with biofilms were placed into a 48-well plate and topically treated by adding treatment solutions (800 μl, 600 μl PMs@NaF-SAP, PMs@NaF, SAP or ddH_2_O and 200 μl culture medium) for 30 min at 37 °C. After each treatment, the discs were washed with sterile PBS three times to remove unbound material and transferred to a new 48-well plate containing BHIS culture medium (800 μl) for further incubation for 24 h. To analyze the antibiofilm effect of PMs@NaF-SAP on the biofilm at the end of the experimental period, after a gentle wash with PBS 3 times to remove unattached bacteria, we performed three similar tests as described above, including CLSM, CFU counting (Fig. S14) and crystal violet staining (Fig. S15). Three independent experiments were conducted, and the data are presented as the mean ± SD.

#### 2.6.7 16 S rRNA PCR analysis of antibacterial mechanism

qRT–PCR was used to explore the antibacterial adhesion and biofilm resistance of PMs@NaF-SAP at the genetic level. The assay was performed by measuring the expression of *S. mutans*-specific targeted genes (gtfB, gtfC, comD, comE, and luxS). The biofilms on HA discs were created as described above in the antibacterial adhesion and biofilm resistance section. Then, the bacterial cells were obtained by centrifugation at 3000*g* for 6 min and washed with PBS three times. RNA was extracted and purified by standard protocols optimized for biofilms (Ultrapure RNA Kit). Afterward, TB Green Premix Ex TaqTM Ⅱ (Tli RNaseH Plus, Takara Bio Inc., Japan) was used for RNA reverse transcription, and quantitative amplification was performed using HiScript III RT SuperMix for qPCR (+gDNA wiper, Nanjing Vazyme Biotech Co., Ltd.). The primers were synthesized by Wuhan Tianyi Huiyuan Bioscience & Technology, Inc., and relevant sequences are listed in Table S1. A standard curve of each primer was used to determine the relative amount of cDNA molecules, and the relative expression was calculated with 16 S rRNA as a reference. PCR results were analyzed with Bio–Rad CFX Manager.

### 2.7 PMs@NaF-SAP demineralization and remineralization assay

#### 2.7.1 PMs@NaF-SAP demineralization assay

To simulate early caries lesions, the prepared enamel windows of 5 × 4 × 1.5 mm^3^ tooth slices were etched with 37 wt % phosphoric acid for 1 min, followed by washing with deionized water for 1 min. The enamel working surface was facing up, and the other surfaces were coated with acid-resistant nail varnish. Then, PMs@NaF-SAP, PMs@NaF, SAP or deionized water (control) was dropped onto the enamel surface. After 30 min, the samples were rinsed with sterile PBS to ensure removal of the unattached material. Then, all enamel windows were immersed in demineralization solution (2.2 mM CaCl_2_, 2.2 mM NaH_2_PO_4_, 0.05 M acetic acid, pH 4.5) at 37 °C for 5 h. After incubation, the enamel samples were sonicated in water for 15 min, rinsed with water and air-dried at room temperature before examination. The enamel surface morphology was qualitatively analyzed using field emission scanning electron microscope (FE-SEM, Zeiss SIGMA, UK) and atomic force microscope (AFM, MultiMode 8, Bruker). The concentrations of minerals Ca and P before and after treatment were measured using inductively coupled plasma-atomic emission spectroscopy (ICP–AES, IRIS Intrepid II XSP, America). Ca/P loss could be obtained upon calculating the enamel exposed area and was expressed as mm^2^ μg^−1^. The mean Ca/P loss was analyzed in each group at the 5% significance level by analysis of variance (ANOVA). Diffraction of x-rays (XRD, XPert Pro, the Netherlands) and Energy dispersive spectroscopy (EDS, Quanta 200 FEG, FEE, Eindhoven, Netherlands) were used to characterize the crystal texture and elementary composition of mineral loss in each group.

#### 2.7.2 PMs@NaF-SAP remineralization assay

Enamel working surfaces were acid etched, immersed in material solutions and rinsed as described above. Then, all tooth samples were immersed in remineralization solution (7 mL, 2.58 mM CaCl_2_·2H_2_O, 1.55 mM KH_2_PO_4_, 1 mg L^−1^ NaF, 180 mM NaCl, 50 mM Tris-HCl, pH 7.6) for 24 h. After incubation, the samples were sonicated in water for 15 min, washed with water and air-dried at room temperature before examination. The enamel surface morphology was qualitatively analyzed by FE-SEM and AFM. The concentrations of minerals Ca and P before and after treatment were measured by ICP–AES. Ca/P gain could be obtained upon calculating the enamel exposed area and was expressed as mm^2^ μg^−1^. The mean Ca/P gain was analyzed in each group at the 5% significance level by ANOVA. XRD and EDS were then used to characterize the crystal texture and elementary composition of mineral deposits in each group.

#### 2.7.3 Mechanical property of PMs@NaF-SAP on tooth enamel

After analyzing the effect of PMs@NaF-SAP on demineralization and remineralization of enamel morphology, the recovery of mechanical properties was also measured. A calibrated Vicker's microhardness testing machine (HXD-2000 TM/LCD; Taiming) was used to measure the SMH of the enamel specimen. For each sample, a load of 50 g for 10 s dwelling was applied, and five indentations were performed at a distance of at least 100 μm. All samples were measured before and after artificial demineralization and remineralization. Surface hardness loss (% SHL) and surface microhardness recovery ratio (% SMHRR) served as indices of mineral loss and gain of enamel, respectively, and were calculated as follows:

(3) % SHL = [(SMH_0_ - SMH_1_)/SMH_0_] × 100%

(4) % SMHRR = (SMH_2_ − SMH_1_)/(SMH_0_ − SMH_1_) × 100%

Where SMH_0_, SMH_1_ and SMH_2_ are the surface microhardness of the enamel sample before demineralization, after demineralization and after remineralization, respectively.

AFM was also used to assess the changes in roughness on enamel surfaces among the treatments.

### 2.8 Cytotoxicity assay *in vitro*

The cytotoxicity of PMs@NaF-SAP was evaluated in MC 3T3 cells by CCK-8 assay. MC 3T3 cells were obtained from the School and Hospital of Stomatology, Wuhan University and were incubated in alpha-modified Eagle's medium (α-MEM) with 10% heat-inactivated fetal bovine serum (FBS) and 1% penicillin–streptomycin at 37 °C with 5% CO_2_ and 95% relative humidity. In the CCK-8 assay, 3T3 cells were seeded in a 96-well plate (n = 27) at a density of 5000 cells per well in α-MEM medium (100 μl). After 24 h of incubation, the media was replaced with fresh medium containing PMs@NaF-SAP or CHX solution (2 mg mL^−1^) at 1/5, 1/10, 1/15, 1/20, 1/30, 1/50 or 1/100 dilution (n = 3 wells per condition with three replicates). Media with or without cells served as the negative group or blank group. After 24 h of incubation, the solution in each well was replaced by CCK-8 (10 μl) in fresh medium (100 μl). The cells were incubated for another 2 h. The absorbance was measured at 450 nm using a microplate reader. The procedure continued, and the absorbance was tested on Days 1, 2, and 3. The cell viability (%) with PMs@NaF-SAP and CHX was computed as 100 × ([A]_treatment_ – [A]_blank_)/([A]_control_ – [A]_blank_). The absorbance was the average data calculated from three wells.

Additionally, the cytoskeleton of 3T3 cells was stained with FITC-phalloidin. CLSM was also performed to observe the morphology of cells exposed to medium containing PMs@NaF-SAP or CHX solution at 1/10, 1/20, 1/30, 1/50 or 1/100 dilution. Cells incubated in medium without materials served as the blank control. Fluorescence intensity measurements were performed for relative quantitative analysis based on the CLSM images by Imaris 7.4.2.

### 2.9 In vivo study

#### 2.9.1 In vivo anti-caries efficacy of PMs@NaF-SAP

Animal experiments were permitted by the School and Hospital of Stomatology, Wuhan University. Thirty female, specific pathogen-free (SPF) Sprague Dawley (SD) rats aged 28 days were used to perform the *in vivo* experiments. To establish a rodent model of dental caries, animals were fed a cariogenic Keyes 2000 diet and 5% sucrose water ad libitum. All rats were prescreened for *S. mutans* free prior to infection by plating on MSB agar, which was selective for the growth of *S. mutans* to avoid contamination by other microbiota in the oral environment. Any *S. mutans*-positive rat was excluded from further experiments. All animal teeth were then topically inoculated with *S. mutans* (100 μl) at mid-logarithmic growth for 7 consecutive days, and another 5 days were allowed to develop infection. At the end of this stage, *S. mutans* infection was confirmed by MSB agar plates. Then, the rats were randomly divided into five treatment groups (n = 6): (1) distilled water (negative control), (2) PMs@NaF, (3) SAP, (4) PMs@NaF-SAP and (5) CHX (positive control). The animals were topically treated daily at 9 a.m. with formulations (100 μl) using a custom-made applicator. The treatment proceeded for 4 weeks. All rats were weighed weekly, and physical appearances were noted daily. At the end of the experiment, oral microbiota samples were collected using sterile swabs, and animals were sacrificed by asphyxiation. The teeth, gums, upper and lower jaws were dissected aseptically and fixed in formalin (4%). The molars were stained with murexide solution (0.4%) for 12 h and prepared for caries scoring according to Keyes’ system. Determination of caries scores was performed by one calibrated examiner and two other examiners. A stereomicroscope (Zeiss, Germany) was used to measure the incidence and severity of smooth-surface and sulcal-surface lesions. High-resolution microcomputed tomography (micro-CT, Super Nova MicroPET/CT, SNPC-303) was used to evaluate the microarchitecture of molars and the carious levels in the teeth.

#### 2.9.2 16 S rRNA gene amplicon sequencing *in vivo*

Dental microbiota samples were stored at −80 °C until DNA extraction, which was performed with the E.Z.N. A™ Mag-Bind Soil DNA Kit (OMEGA, M5635-02) according to the manufacturer's instructions. DNA was then sequenced at Sangon Biotech (Shanghai) Co., Ltd. using primers targeting the V3 and V4 regions of the 16 S rRNA gene. The primer sequences were as follows: V3–F 5′-CCTACGGGNGGCWGCAG-3′, V4-R 5′-GACTACHVGGGTATCTAATCC-3′. Sequences were clustered into operational taxonomic units (OTUs) at a 97% similarity threshold using Usearch version 11.0.667 [34,35]. We used R version 3.6.0 for statistical analysis and data processing [36]. Richness, diversity, and bacterial taxon abundances were compared using mothur version 1.43.0 [37]. To assess differences in the relative abundance of bacterial taxa, we used a *t*-test of log-transformed relative abundance [38]. To assess differences between groups, we used relative abundances, principal coordinate analysis (PCoA) and the Anosim test [39].

#### 2.9.3 Histopathological analysis

Both gingival and palatal tissues were collected and processed for hematoxylin and eosin (H&E) staining for histopathological analysis.

### 2.10 Statistical analysis

At least three independent experiments were performed for each experiment. All results are presented as the mean ± standard deviation. GraphPad Prism 8 and Origin 2019 software were used to perform statistical analyses, as shown in the figure captions. Statistical significance between groups was examined using one-way ANOVA with Tukey's multiple comparison test, accepting significance at p < 0.05. Specific tests are denoted in the figure legends.

## 3 Results and discussion

### 3.1 Synthesis and characterization of PMs@NaF-SAP

**Fig. 2:**
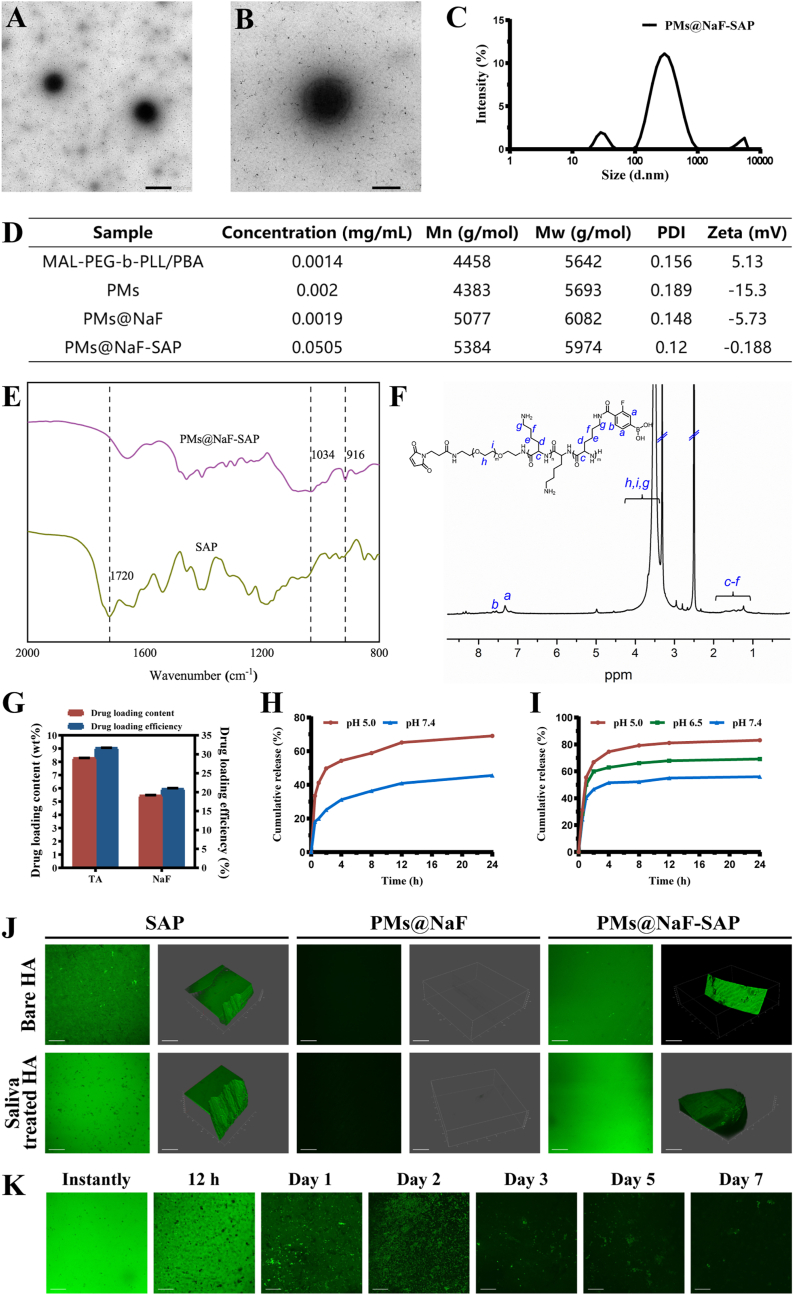
Characterization of PMs@NaF-SAP and its binding to the enamel surface. (A) and (B) TEM images of PMs@NaF-SAP (scale bar for A: 500 nm, B: 200 nm). (C) Size distribution of PMs@NaF-SAP determined by DLS. (D) Concentrations, molecular weights (Mn and Mw), PDI and zeta potentials of stage products. (E) FT-IR spectra of SAP and PMs@NaF-SAP. (F) ^1^H NMR of MAL-PEG-*b*-PLL/PBA in DMSO-d_6_. (G) Drug loading content (%) and efficiency (%) from PMs@NaF-SAP, the results were reported as mean ± s.d (n = 3). Cumulative *in vitro* release of (H) TA and (I) NaF from PMs@NaF-SAP at different pH values. (J) Adsorption capacity assay and (K) adsorption time assay of PMs@NaF-SAP when monitoring the oral salivary environment (scale bar: 50 μm).

PMs@NaF-SAP was synthesized via the self-assembly of MAL-PEG-*b*-PLL/PBA and TA in deionized water containing NaF, followed by conjugation with SAP. The entire synthetic process is detailed in Fig. 1 and Fig. S1. Briefly, MAL-PEG-*b*-PZLL was synthesized via ring-opening polymerization. MAL-PEG-*b*-PLL was prepared by benzyl deprotection of MAL-PEG-*b*-PZLL. MAL-PEG-*b*-PLL/PBA was synthesized by conjugating FPBA to the pendent amino group of PLL. Polymeric micelles are a type of nanomaterial with a well-defined core-shell structure composed primarily of amphiphilic block single-chain polymers, typically consisting of PEG, which provides advantages for nanocarriers to penetrate within biofilms [[40], [41], [42]]. The characterization of MAL-PEG-*b*-PLL/PBA is described in Fig. 2 and Fig. S2. As shown in Fig. 2D, the number average molecular weight (Mn) of MAL-PEG-*b*-PLL/PBA was 4458 g mol^−1^, and the weight average molecular weight (Mw) was 5642 g mol^−1^, which were measured by GPC. According to ^1^H NMR, the PEG linker exhibited large peaks at 3–4 ppm, and the peaks at approximately 7.3 ppm were characteristic of the phenylene protons of FPBA, indicating that FPBA was successfully conjugated with polymers (Fig. 2F).

The side effects of current conventional antimicrobials in clinical therapy, such as alterations in taste and extrinsic tooth staining caused by CHX, cannot be neglected. TA is a water-soluble natural polyphenol that can be extracted from fruits, tea, red wine, etc [43]. Due to its abundant polyphenolic groups, TA has been reported to exhibit excellent antibacterial properties by destroying bacterial stability, increasing the permeability of the cell membrane and inhibiting enzymes [44]. Thus, incorporating TA into nanoparticles will endow it with good antimicrobial properties, and the catechol groups in TA enable the formation of pH-cleavable boronic ester bonds between PBA and TA, which self-assemble into the PMs structure and make it bacteria-responsive toward an acidic caries microenvironment. NaF, which was introduced over 60 years ago, has been recognized as a key and widespread contributor to the prevention and suppression of caries by practicing professionals and dental researchers worldwide [8,45]. Fluoride functions by reducing the solubility of enamel, inhibiting the growth and acid production of cariogenic bacteria, affecting the morphology and structure of teeth and promoting their remineralization, which have been proven by a series of studies over many decades [17,46,47]. Therefore, we incorporated NaF as a restorative agent into the bacteria-responsive system. We hypothesized that the multidrug-loaded, bacteria-responsive PMs@NaF could be incorporated into biofilms and exert its bactericidal and restorative activity via accurately controlled drug release while exerting localized remineralization effects.

Therefore, we designed a type of MAL-PEG-*b*-PLL/PBA-based PMs loaded with TA and NaF. TA is conjugated to PBA through phenylboronic acid-catechol interactions, thus forming the boric acid ester bond, which is pH-sensitive and undergoes acid cleavage under cariogenic acidic conditions [48]. NaF is co-loaded into the micelles through physical encapsulation. Thus, chemical conjugation and physical encapsulation are applied for multidrug loading into micelles and endow them with bacterial responsiveness. TA is negatively charged at physiological pH and can interact electrostatically with positively charged PLL to form the classic core-shell structure of micelles. In addition, the presence of PEG and its negatively charged characteristics at physiological pH enable micelles to penetrate biological membranes to transport drugs.

To overcome the rapid clearance of drugs from the oral cavity, it is highly desired to identify an ideal anchor like the dental plaque that can make nanoparticles specifically attached onto hydroxyapatite (HA, the primary component of tooth enamel) [49]. Salivary acquired pellicle is a thin film rapidly formed by salivary proteins on the surface of teeth [32,33]. Because they are rich in some salivary proteins, particularly statherin, the calcium-binding domains endow the proteins selectively adsorb onto HA in seconds to a couple of minutes [[50], [51], [52]]. The N-terminal hexapeptide sequence DpSpSEEK is the primary domain of statherin that is fixed on the surface of HA [53]. Considering the conjugation of the peptide with PMs@NaF, we attach cysteine to the end of the sequence DpSpSEEK, which allows it to form a thioether conjugate with the above MAL group via a maleimide sulfhydryl reaction and thus obtain the final product PMs@NaF-SAP. Combined with the plaque-inspired strategy, the bacteria-responsive controlled release system is expected to produce biomaterials with strong interfacial adhesion and solve the problem of long-term retention of drugs in the oral cavity.

Characterizations of PMs@NaF-SAP and stage products are described in Fig. 2 and Figs. S2–S6. A uniform spherical morphology of PMs@NaF-SAP was confirmed by TEM (Fig. 2A and B), which indicated a classic spherical core-shell structure of micelles. No marked changes in the size or morphology of PMs@NaF were observed after conjugation with SAP (Fig. S5). The particle diameter revealed relatively narrow size distributions (PDI = 0.12) for PMs@NaF-SAP with an average diameter of approximately 300 nm (Fig. 2C), corresponding to the TEM result. The structure, composition, and physical properties of polymers synthesized in each procedure are shown in Fig. 2D. Successful conjugation was confirmed by FTIR (Fig. 2E). There was a small peak at 1720 cm^−1^ in the spectrum of SAP, which corresponded to the thiol group of cysteine. For the spectra of PMs@NaF-SAP, the thiol band disappeared and two new peaks were generated at 1034 and 916 cm^−1^. These new extensions appeared during the production of thioether groups that were attached to the conjugation between SAP and PMs@NaF, which demonstrated a successful connection. The ^1^H NMR result was in accordance with the FTIR data, where the chemical shifts at approximately 6–7 ppm demonstrated that the acryloyl group was introduced to TA, indicating the construction of PMs loaded with TA (Fig. 2F and Fig. S3).

The dual-drug loaded PMs were further investigated for drug encapsulation. As shown in Fig. 2G, the DLC of NaF and TA in PMs@NaF-SAP was 5.5% and 8.3%, respectively. The corresponding DLE were 20.9% and 31.7%, respectively. Results showed that the micelles successfully loaded the two drugs concurrently, and there were no large differences in PMs@NaF with or without conjugated SAP (Fig. S7). Additionally, micelles tend to load hydrophobic drugs into the core, so as that the loading rate of NaF is not too high because it is only physically encapsulated into the micelles, which also corresponds to the results we obtained.

The pH-sensitive boronate ester formed by PBA and TA laid the foundation for the controlled release of PMs, which were highly sensitive and endowed PMs intelligent drug delivery systems. When caries occurs along with acidification, the boronate ester is cleaved, followed by destabilization of the micelles, contributing to the rapid release of TA and NaF. To confirm this, *in vitro* drug release from micelles was investigated under different pH conditions. First, we monitored TA release by HPLC since it had a characteristic absorption peak at 276 nm. The release rate of TA at pH 5.0 was several times faster than that at pH 7.4 for both PMs@NaF and PMs@NaF-SAP, which can be attributed to the pH cleavability of phenylboronate (Fig. 2H and Fig. S8). Also, nearly 70% of TA was cumulatively released from PMs@NaF-SAP within 24 h at pH 5.0, compared to only approximately 40% at pH 7.4. These results confirmed the high stability of PMs and implied their acid cleavable behavior.

Based on the pH responsiveness of PMs, NaF should also be controllably released from micelles. We investigated three different pH conditions to simulate NaF release during changes in the caries’ microenvironment. As expected, more cumulative release and a faster rate of NaF were observed under more acidic pH conditions regardless of whether SAP was conjugated (Fig. 2I and Fig. S9). The release of NaF from PMs@NaF-SAP could be sustained within 24 h, and reached approximately 80% of the cumulative release at pH 5.0. From similar trends in TA and NaF release, we found a large burst release within the first 2 h and a long-term release over a time period of 24 h. These results suggested the successful construction of an intelligent drug delivery system where the release of TA and NaF from PMs@NaF-SAP was selective and accelerated in response to acidic pH. Slow release of drugs at normal physiological pH indicated the high stability of PMs@NaF-SAP in the healthy oral cavity, which reduced drug loss and side effects. Conversely, the sufficient and rapid release at acidic cariogenic pH ensures targeted drug delivery and highly efficient therapeutic effects.

Next, the adsorbability of PMs@NaF-SAP to human enamel was verified. Human enamel samples were immersed in Fam-labeled SAP, PMs@NaF and Fam-labeled PMs@NaF-SAP solution separately. Fig. 2J shows the green fluorescence distribution on both Fam-labeled SAP- and Fam-labeled PMs@NaF-SAP-coated surfaces, while there was no fluorescence on the PMs@NaF coated surface. Results demonstrated that PMs@NaF-SAP could effectively adsorb on enamel slices. We then investigated the adsorption time of PMs@NaF-SAP to enamel samples in the mimetic flowing saliva environment. As shown in Fig. 2K, there was still a large part of PMs@NaF-SAP adsorption on enamel surfaces after 2 days of incubation, indicating that the adsorbability of PMs@NaF-SAP could tolerate the buffering effect of saliva to a large extent, which was important for oral clinical applications.

### 3.2 Antibacterial adhesion properties and anti-cariogenic biofilm abilities *in vitro*

**Fig. 3:**
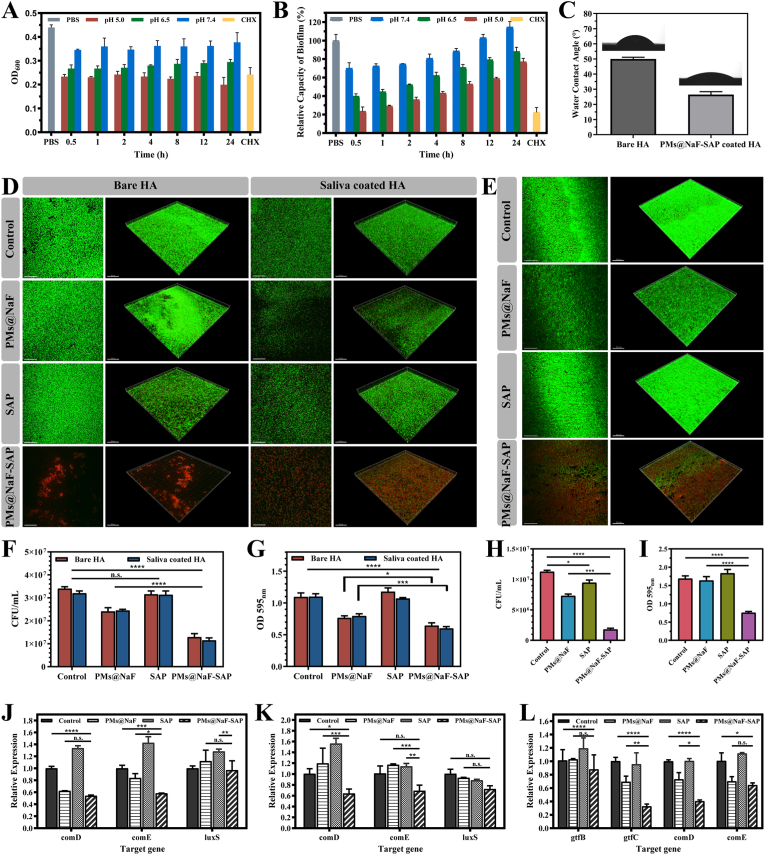
Antibacterial adhesion abilities and cariogenic biofilm resistance of topical PMs@NaF-SAP treatment *in vitro*. (A) OD_600_ value of *S. mutans* and (B) relative capacity of biofilm (%) as a function of incubation with PMs@NaF-SAP at pH 7.4, 6.5 or 5.0, with CHX as positive control and PBS as blank control. (C) Water contact angle (°) of bare HA and PMs@NaF-SAP coated HA. (D) Adhesion of *S. mutans* on HA and sHA discs evaluated by CLSM (live cells are labeled in green by SYTO 9 and dead cells in red by propidium iodide, scale bar: 50 μm), (F) CFU counting and (G) crystal violet staining after pre-treated with sterile PBS (control), PMs@NaF, SAP or PMs@NaF-SAP solutions. (E) CLSM images (scale bar: 50 μm), (H) CFU counting and (I) crystal violet staining evaluating the penetration and antibiofilm activity of sterile PBS (control), PMs@NaF, SAP or PMs@NaF-SAP solution treating on the pre-established *S. mutans* biofilm. Relative expressions of genes related to *S.mutans* (J) on HA or (K) sHA when treated with sterile PBS (control), PMs@NaF, SAP or PMs@NaF-SAP in antibacterial adhesion assay and (L) in cariogenic biofilm resistance assay, respectively. Each experiment was performed in triplicate, and the data were presented as mean ± s.d. (n = 3). The quantitative data were subjected to Student's *t*-test for a pairwise comparison. *p < 0.5, **p < 0.01, ***p < 0.001, ****p < 0.0001.

Antibacterial activity is one of the most important properties for oral nanomaterial application, allowing for caries etiology. To examine the antibacterial activity at different pH values, PMs@NaF-SAP was incubated with planktonic cells of *S. mutans* for 24 h. The antibacterial ratios of PMs@NaF-SAP were investigated via OD_600_, which was used to monitor bacterial growth and viability. As shown in Fig. 3A, PMs@NaF-SAP exhibited better performance against *S. mutans* at lower pH, which was primarily attributed to improved drug release under acidic conditions. The antibacterial capability of PMs@NaF-SAP at pH 5.0 may have an advantage over the positive control treatment with CHX.

The formation of oral biofilms plays a critical role in antimicrobial resistance and caries progression. Thus, we assessed the relative capacity of biofilm inhibition under different pH conditions when treated with PMs@NaF-SAP using a preestablished biofilm model that mimicked the cariogenic microenvironment. Similarly, the OD_600_ was applied to monitor the development and activity of the biofilm. The degree of reduction in biofilm formation compared to untreated cells was defined as the relative capacity of biofilm (%). The amount of biofilm formed for the blank control was defined as 100%. The biofilm treated with PMs@NaF-SAP was markedly inhibited when compared to the control, with the effect existing up to 24 h. More acidic conditions encountered, more antibiofilm activity was observed (Fig. 3B).

Next, contact angle measurements were performed to detect the hydrophilicity and hydrophobicity of the materials. Untreated control HA discs were highly hydrophobic with a contact angle of approximately 50°, and the droplet of ddH_2_O remained in a semispherical form (Fig. 3C). In contrast, PMs@NaF-SAP treatment resulted in a hydrophilicity change in enamel wettability to ddH_2_O, with a contact angle of approximately 25°, which prevented bacterial adhesion to enamel surfaces [54,55].

To further evaluate these effects *in vitro*, we tested the antibacterial ability and cariogenic biofilm resistance. In the antibacterial adhesion study, four different tests were conducted on the PMs@NaF-SAP-coated HA discs to determine whether PMs@NaF-SAP could inhibit bacterial adhesion. First, confocal fluorescence imaging was used to visualize bacterial adhesion upon live/dead bacterial staining. Live cells were dyed green, while dead cells were dyed red. A remarkable inhibitory effect on *S. mutans* adhesion was observed with PMs@NaF-SAP compared to PMs@NaF, SAP alone or the control (Fig. 3D). No significant differences were detected between HA slices with or without saliva treatment. This result was supported by quantitative fluorescence intensity analysis, where we examined a much lower bacterial count and a much higher ratio of dead bacteria (Fig. S10). Additionally, a slight decrease in the number of bacteria was observed in the SAP group, indicating a certain degree of antibacterial adhesion properties. Also, CFU counting and crystal violet staining can be important parameters for biofilm biomass disruption to determine antibiofilm efficacy. As shown in Fig. 3F and G, PMs@NaF-SAP reduced bacterial biomass more than other formulations, indicating the potent ability of impairing *S. mutans* accumulation and preventing biofilm formation. Thus, all assessments verified the strong resistance of PMs@NaF-SAP to bacterial and biofilm adhesion.

Next, regarding cariogenic biofilm resistance, to determine whether PMs@NaF-SAP can penetrate into the biofilm and kill the bacteria within it, we developed a cariogenic biofilm on saliva-coated hydroxyapatite (sHA) discs and then administered topical treatments. CLSM images of HA discs treated with PMs@NaF, SAP alone or the control showed more live *S. mutans* than PMs@NaF-SAP (Fig. 3E), which was consistent with a marked increase in the number of dead bacteria in the fluorescence intensity analysis (Fig. S13). When treated with PMs@NaF alone, some antibacterial activity was assessed, albeit to a much lower degree than for PMs@NaF-SAP. We then used BHI agar culture for CFU counting. PMs@NaF-SAP potently resisted established biofilm viability by ∼1 × 10^7^ CFU mL^−1^, while PMs@NaF or SAP alone resulted in only an ∼4 × 10^6^ or ∼2 × 10^6^ CFU mL^−1^ reduction, respectively (Fig. 3H). In terms of crystal violet staining, treatment with the control, PMs@NaF and SAP showed minimum effects against *S. mutans* similarly, while PMs@NaF-SAP exhibited an effective antibiofilm capability (p < 0.0001) (Fig. 3I). These results thus demonstrated the biofilm targeting specificity of PMs@NaF-SAP, which could effectively resist bacterial adhesion, penetrate into and kill bacterial cells within cariogenic acidic biofilms. Topical application of drugs suffers from poor penetration of biofilm matrices, where the presence of extracellular polymeric substances with their altered microenvironment reduces drug access [[56], [57], [58]]. Nanoparticle drug delivery systems protect drugs from pH degradation in harsh biofilm niches, and meanwhile exploit the unique microenvironment for stimuli-responsive drug release [59]. The chemical and material structure of the micelles we used here provide excellent nanoparticle properties, including size, shape, surface functionalization, and drug-loading properties for robust antibiofilm efficacy against bacteria.

Additionally, we explored the antibacterial mechanism behind the proposed material. 16 S rRNA PCR was performed to evaluate the expression of genes associated with biofilm formation (gtfB/C) and the QS system (comD/E and luxS) of *S. mutans* UA159 treated with PMs@NaF-SAP. Similar HA disc models were used to establish and generate biofilms for gene expression analysis. As shown in Fig. 3J and K, regardless of whether HA discs were pretreated with saliva, the expression of all biofilm-related and QS system-associated genes was dramatically downregulated by PMs@NaF-SAP. Similar trends were assessed in antibiofilm tests, which agreed with its ability to resist biofilm formation and inhibit the QS system (Fig. 3L). The addition of PMs@NaF also showed certain effect on gene expression in the antibiofilm test, while SAP alone had little effect comparable to the control group.

Based on all the results above of the antibacterial and antibiofilm tests, we found that the use of PMs@NaF alone on the tooth enamel could not prevent bacterial adhesion because it had no adhesion effect on HA. It showed a weak antibacterial effect when applied after biofilm formation, while the buffering effect of saliva made it fail to remain on the enamel surface to exert its medicinal effect for a long time. The use of SAP alone theoretically had a certain ability to resist bacterial adhesion since it was one of the components of salivary proteins, however, it had no effect on penetrating into the biofilm and killing bacteria within it.

### 3.3 Restorative remineralization and mechanical recovery properties of PMs@NaF-SAP on tooth enamel

**Fig. 4:**
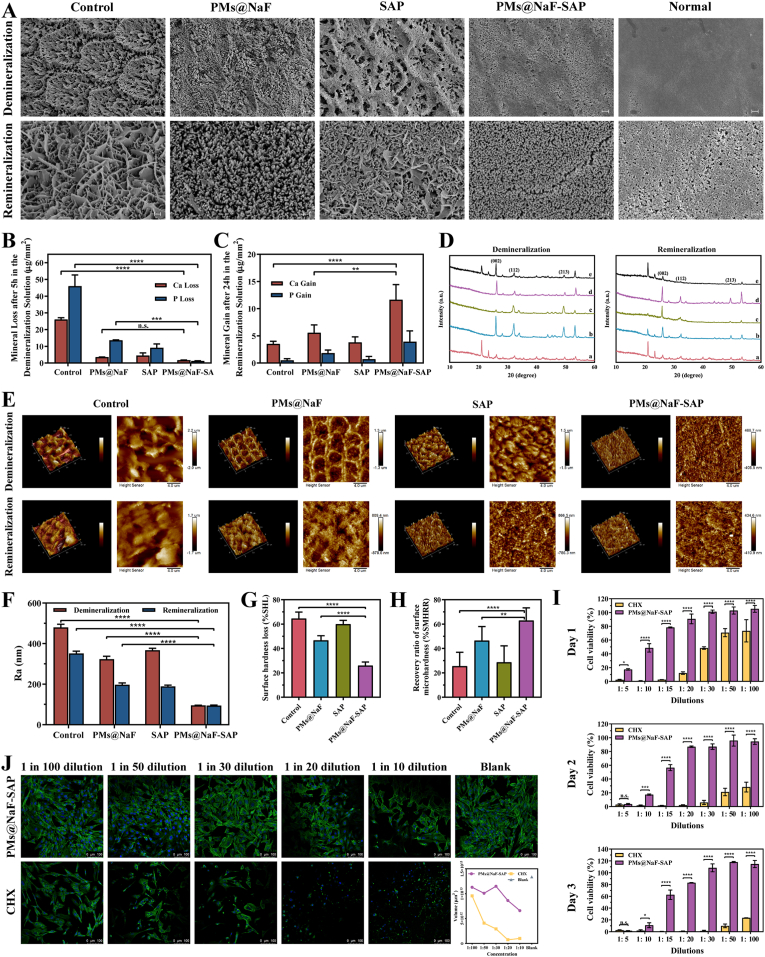
Demineralization and remineralization effects, mechanical recovery properties and cytotoxicity of PMs@NaF-SAP. (A) FE-SEM micrographs of the demineralization and remineralization surfaces in sterile PBS (control), PMs@NaF, SAP or PMs@NaF-SAP and normal enamel (scale bar: 1 μm). (B) Amount of Ca/P loss from enamel surfaces in demineralization solutions measured by ICP-AES after sterile PBS (control), PMs@NaF, SAP or PMs@NaF-SAP coating. The red bar denotes Ca loss and the blue bar indicates P loss. (C) Amount of Ca/P gain of enamel surfaces after 24 h in remineralization solutions by ICP-AES after sterile PBS (control), PMs@NaF, SAP or PMs@NaF-SAP coating. The red bar indicates the Ca gain and the blue bar denotes the P gain. (D) The XRD spectra for the mineral phase evolution on the demineralization or remineralization enamel window: the normal enamel (line a), sterile PBS coated (line b), PMs@NaF coated (line c), SAP coated (line d) and PMs@NaF-SAP coated (line e); (E) AFM images (scale bar: 4 μm) and (F) R_a_ of demineralization or remineralization surfaces in sterile PBS (control), PMs@NaF, SAP or PMs@NaF-SAP. (G) The % SHL of the four groups after demineralization. (H) The % SMHRR of the four groups after remineralization. (I) Cell viability of MC3T3 cells at various dilutions of medium containing PMs@NaF-SAP or CHX by CCK-8 assay. (J) Cytoskeletion staining and fluorescence quantitative analysis of MC 3T3 cells at various dilutions of medium containing PMs@NaF-SAP or CHX, with cells untreated as the blank control (scale bar: 100 μm). Each experiment was performed in triplicate, and the data were presented as mean ± s.d. (n = 3). The quantitative data were subjected to Student's *t*-test for a pairwise comparison. *p < 0.5, **p < 0.01, ***p < 0.001, ****p < 0.0001.

Several assays were performed to investigate the demineralization inhibition and remineralization promotion capacities of PMs@NaF-SAP. After enamel slices were exposed to the demineralization solutions for 5 h, SEM showed that the control samples exhibited a classic fish scale structure, where the glaze pillars and the structure between the glaze pillars and the glaze sheath were all dissolved. Treatment with SAP alone showed similar results to the control, while PMs@NaF alone exhibited a limited effect to a certain extent resulting from unavailability for retention on enamel. Conversely, PMs@NaF-SAP group presented a relatively smooth surface, and its enamel prism structure was primarily sheltered, indicating its excellent ability to inhibit demineralization (Fig. 4A). ICP–AES was also used for quantitative evaluations. As shown in Fig. 4B, the loss of Ca and P from PMs@NaF-SAP-coated enamel surfaces were significantly reduced compared with the control group (p < 0.0001), while PMs@NaF-coated and SAP-coated enamel decreased as well to some extent. PMs@NaF-SAP thus demonstrated a stronger ability to inhibit demineralization.

After 24 h of exposure to the remineralized solution, SEM showed the formation of new HA crystals on the demineralized enamel surfaces of all four groups (Fig. 4A), but their morphologies were distinguished from each other. The microstructure of the regenerated crystal treated with PMs@NaF or PMs@NaF-SAP appeared to be acicular and had an ordered nanorod structure morphologically similar to the natural enamel but was flaky and distributed randomly in the SAP and control groups. In particular, the crystals of PMs@NaF-SAP group aggregated in a much thicker, longer and denser way, indicating a faster mineralization speed and better regulation of the new HAP crystal growth on acid-etched surfaces. Based on the ICP–AES calculations, significant gains in Ca and P were assessed in the PMs@NaF-SAP group compared with the control group (p < 0.0001, Fig. 4C). Also, PMs@NaF treated group showed a marked difference from the PMs@NaF-SAP group (p < 0.01), suggesting the importance of the specific adsorption to enamel for promoting remineralization of PMs@NaF-SAP.

Further assessments were performed to confirm whether the regenerated crystals were hydroxyapatite crystals. XRD was used to characterize the crystalline structure (Fig. 4D). All samples showed characteristic diffraction peaks of hydroxyapatite, however, the relative intensity of different peaks varied between samples, demonstrating the different crystal orientations after demineralization and remineralization. The results were consistent with the SEM images, which showed that crystal orientation after remineralization by PMs@NAF-SAP was more closely resembled to the normal enamel. EDS spectra of samples before and after treatment were assessed (Fig. S17). The calcium-phosphorus atomic ratio was 1.47 for normal enamel. The ratios for the control, PMs@NaF, SAP and PMs@NaF-SAP groups after demineralization were 1.40, 1.42, 1.43 and 1.47, while were 1.48, 1.48, 1.43 and 1.52 after remineralization, respectively, indicating that mineral deposits in all four groups after treatment had similar Ca–P atomic ratios to normal enamel.

To evaluate the mechanical property of the demineralized and remineralized enamel treated with PMs@NaF-SAP, the micro Vickers hardness test and AFM test were used to measure the microhardness and roughness, respectively. AFM can be used to describe basic three-dimensional patterns and estimate the average mean roughness (R_a_) of the samples, which was used to assess the mean surface roughness. As shown in Fig. 4E, the enamel surface treated with PMs@NaF, SAP alone or the control became more asperous than that treated with PMs@NaF-SAP after acid etching, with the enamel prism structure exposed, demonstrating its ability to prevent demineralization and maintain the integrity of enamel structure. For the PMs@NaF- and PMs@NaF-SAP-treated samples after remineralization, the newly regenerated crystals on acid-etched enamel appeared to be oriented and uniform bundles of nanorod HAP. The layer and crystals of the PMs@NaF-SAP group were thicker and denser than those of the PMs@NaF group, which were attributed to the effective biomineralization property of PMs@NaF-SAP in situ (Fig. 4F). This result demonstrated that the regrown crystals were uniformly arranged due to the potent effect of PMs@NaF-SAP, making the enamel surface smoother than that of the control group.

Additionally, the amount and microstructure of lost and regenerated minerals play a critical role in determining the average hardness. Fig. 4G and H shows the surface microhardness via surface hardness loss (% SHL) and surface microhardness recovery ratio (% SMHRR). No significant differences were found between the SAP and control groups, while the PMs@NaF-SAP group had a markedly lower % SHL and much higher % SMHRR than all other groups, which revealed the least mineral loss and damage to the enamel structure toward demineralization and the potent microhardness recovery after remineralization.

### 3.4 Bioactivity assessment of PMs@NaF-SAP

In addition, we investigated the cell viability to ensure the safety of PMs@NaF-SAP. The cytotoxicity of PMs@NaF-SAP to MC 3T3 cells was investigated using a CCK-8 assay with 0.4% CHX as the positive control. Then, cell viability was found to be greater than 80% at dilutions of medium containing PMs@NaF-SAP ranging from 1:20–1:100. At each dilution, CHX exhibited much higher cytotoxicity than PMs@NaF-SAP (Fig. 4I). Cytoskeletion staining was also used to visualize the cell morphology and activity. According to the CLSM images, 3T3 cells treated with PMs@NaF-SAP in all groups exhibited excellent biocompatibility with a healthy cell morphology and showed no significant difference from the blank control, confirming when the dilution ranged from 1:30–1:100, the proliferation of 3T3 cells was not affected by PMs@NaF-SAP. The CHX-treated cells exhibited an uneven distribution and an irregular, disheveled and shrunken morphology, which was consistent with previous results. Fluorescence quantitative analysis showed similar results (Fig. 4J).

The various components of the nanosystem, including PEG, TA and NaF, are recognized to have good biological safety and have even been incorporated into clinical use [9,60,61]. Conversely, the positive control CHX, as a commonly used oral antibacterial agent in clinical practice, produces certain side effects based on the results above [62,63]. Thus, PMs@NaF-SAP exhibited excellent bioactivity, which showed a greater advantage over CHX and is therefore expected to be a promising application for clinical transformation.

### 3.5 In vivo efficacy of PMs@NaF-SAP

**Fig. 5:**
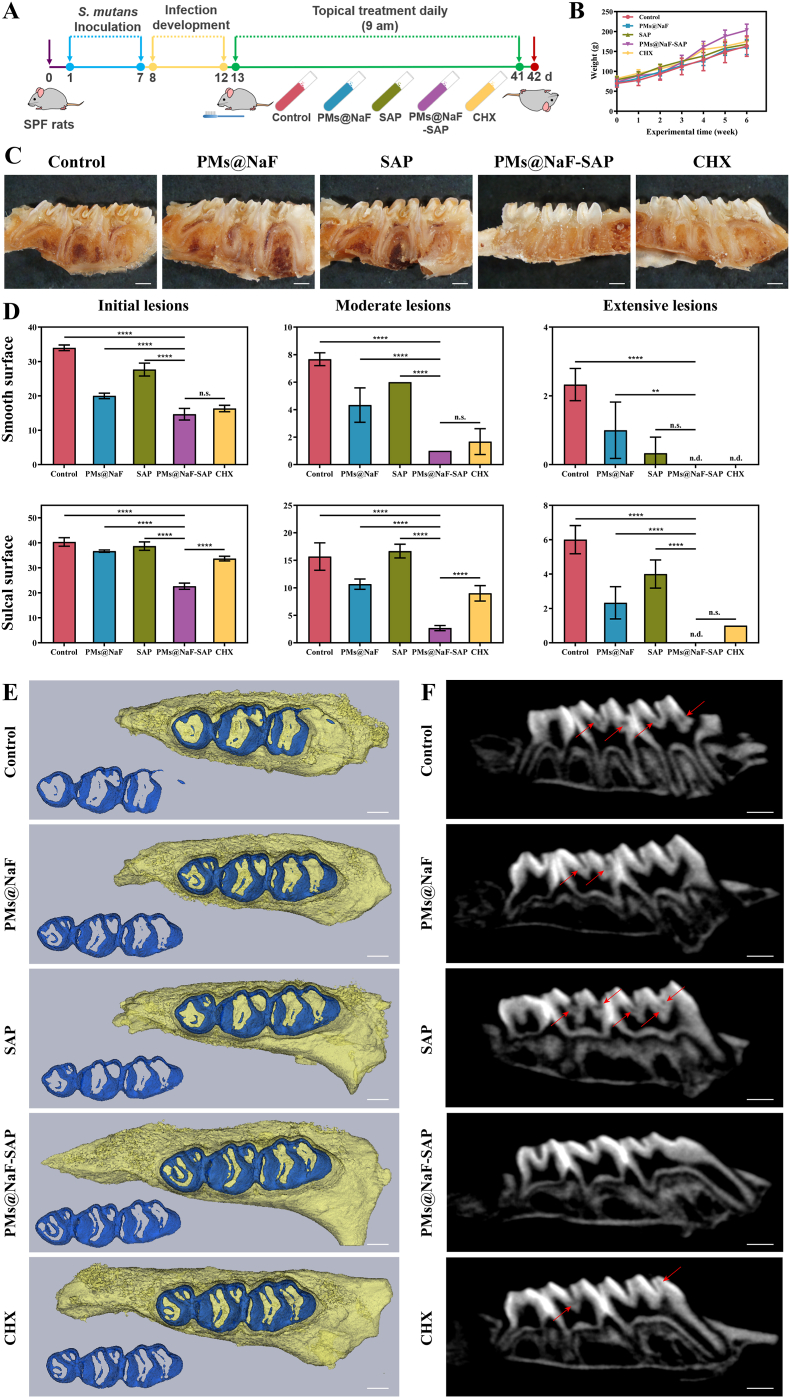
Therapeutic efficacy of topical PMs@NaF-SAP treatment against tooth decay *in vivo* as evaluated by Keyes' scoring and micro-CT analysis. In this model, tooth enamel progressively develops caries lesions (similar to those observed in humans), proceeding from initial lesions of demineralization to moderate lesions and on to extensive lesions characterized by enamel structure damage and cavitation. (A) Experimental design and treatment regimen of the animal model, the treatments (control, PMs@NaF, SAP, PMs@NaF-SAP and CHX) are represented in the centrifuge tubes. (B) Body weights of rats during the experimental period. (C) Images of rat teeth after treatment of the five groups (scale bar: 1 mm). (D) Caries onset and severity of smooth and sulcal surfaces. Caries scores are recorded as stages and extent of carious lesion severity according to Larson's modification of Keyes' scoring system. Data were presented as the mean ± s.d. (n = 6); ordinary one-way ANOVA, p value < 0.01, 0.001 and 0.0001 are indicated by **, *** and ****, respectively; n.s. nonsignificant; n.d. nondetectable. (E) Three-dimensional reconstruction of micro-CT images of maxillary molars in five groups, separated enamel (blue) by setting the density threshold above 4500 Hounsfield units (scale bar: 1 mm). (F) 2D scale sagittal images of the maxillary molars analyzed by micro-CT (red arrows, caries lesion sites, scale bar: 1 mm).

To validate the *in vivo* inhibition of dental caries and restoration effect, we preestablished a cariogenic rodent model according to the plan shown in Fig. 5A. All animals remained in good health until the experimental endpoint and gained weight equally without significant differences between groups (p > 0.05, Fig. 5B). To visually observe the therapeutic effect, we took photographs of general and incisional examples of molars in treated rats after 0.4% murexide staining (Fig. 5C and Fig. S18). Treatment with sterile water (control) or SAP alone had no marked effect on the prevention and treatment of caries in rats. PMs@NaF group showed a relative effect to some extent, while PMs@NaF-SAP performed more efficiently than CHX. The results of Keyes’ scores differed in how much and how severely different treatments caused smooth surfaces or sulcal surfaces of molars (Fig. 5D). As expected, the control group corresponded to the most severe caries, which showed no large differences from the SAP group in initial and moderate lesions. Treatment with PMs@NaF-SAP attenuated the onset and severity of lesions on both smooth surfaces and sulcal surfaces compared with the control, SAP or PMs@NaF alone (p < 0.0001). The efficacy of PMs@NaF-SAP treatment was significantly higher than that of the current gold standard oral antimicrobial agent CHX, particularly in initial and moderate lesions of the sulcal surface (p < 0.0001), showing a potential therapeutic effect of controlling tooth decay. Enamel demineralization induced by acid-producing bacteria is an important feature of tooth decay, which can also be visualized by micro-CT as a complementary method to assess carious lesions. Representative three-dimensional images of maxillary molars for the five groups are shown in Fig. 5E. The teeth in the PMs@NaF-SAP group had a more intact volume and higher mineral density of enamel (blue) than those in the other control groups, which were stripped and reconstructed from the maxillary molars. Also, the enamel treated with PMs@NaF-SAP had fewer demineralized sites (shaded by red arrows) according to the corresponding sagittal section images (Fig. 5F). However, the number and area of sulcal demineralization sites in the control group were more extensive, even reaching the pulp cavity, which indicated that the tooth tissue in these groups was severely damaged. Results showed that PMs@NaF-SAP was efficient at preventing the occurrence and development of tooth decay *in vivo*. We attribute these results to the strong binding to hydroxyapatite, efficient antibacterial and restorative ability of PMs@NaF-SAP in the acidic biofilm microenvironment, thus inhibiting demineralization and promoting remineralization.

### 3.6 Effects of topical PMs@NaF-SAP on oral microflora and soft tissue

**Fig. 6:**
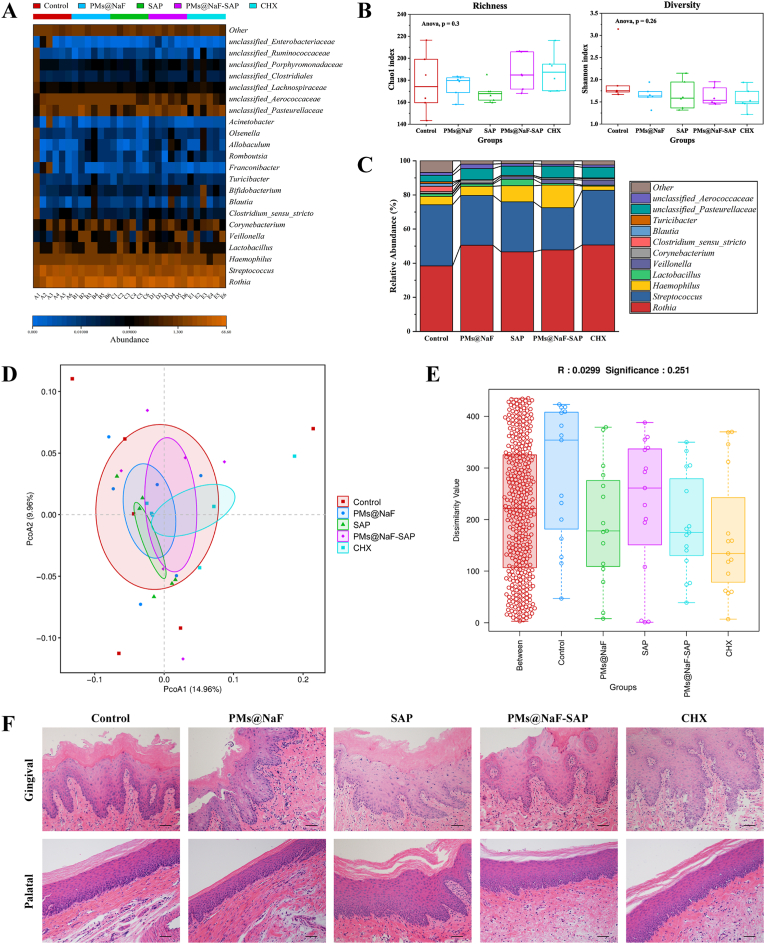
Effects of topical PMs@NaF-SAP on oral microflora and soft tissue *in vivo* after 28 days of treatment (n = 6 per group). **(**A) The heatmap indicated main bacterial genera found across all samples according to treatment groups. (B) Chao 1 richness index and Shannon α-diversity index. The whiskers (bars from the box) represent the upper and lower quartiles of the data. (C) Bacteria relative abundances within rodent body sites for each treatment group. (D) Weighted Unifrac PCoA, which indicated the β-diversity in each group; the analysis revealed that the PMs@NaF-SAP group has similar composition between samples. (E) Bray-Curtis ANOSIM test had R value close to 0 and p value > 0.05, which indicated that there were no significant differences between and within groups based on their Bray–Curtis distances. (F) Histopathology of gingival and palatal tissues in rats treated with PMs@NaF-SAP was similar to control (scale bar: 50 μm).

To further assess the biocompatible effects of topical treatment on the oral microbiota and surrounding soft tissues after 28 days, analyses of microbiome sequencing and histopathological images on gingival and palatal tissues are shown in Fig. 6 and Figs. S19–20. All treatment groups revealed similar oral microbial compositions (Fig. 6A). The Chao 1 richness index and Shannon diversity index showed that neither richness (number of bacterial species) nor diversity (distribution of bacterial species) of the treatment groups were affected (Fig. 6B; p > 0.05 by Wilcoxon rank sum test), indicating that the proposed treatments did not disrupt the ecological balance of the oral microbiome. Using relative abundances (Fig. 6C), we investigated the dominant bacterial composition and their relative abundance ratio in each treatment. While treatment with PMs@NaF-SAP comprised the lowest proportion of *Streptococcus*, oral sites by the *Rothia* and *Streptococcus* genera exhibited a relative abundance of approximately 25%. Also, weighted UniFrac distances analyzed by PCoA in the treatment groups suggested that PMs@NaF-SAP group had a similar composition between samples within the group and was most similar to the species composition of the control group (Fig. 6D). In addition, the ANOSIM test, which served to determine whether the grouping was meaningful, indicated that the R value was near 0, and the p value was >0.05 (Fig. 6E), which verified that there were no statistically significant differences between and within groups. The lack of impact on oral microbial composition and diversity is likely because PMs@NaF-SAP has increased bactericidal activity at acidic pH values present in acidogenic biofilms caused by cariogenic bacteria such as *S. mutans*.

Histopathological analysis of gingival and palatal tissues was also conducted to assess the safety and biocompatibility of PMs@NaF-SAP *in vivo* (Fig. 6F). No significant signs of adverse effects, such as inflammatory responses, proliferative changes, tissue damage or necrosis, were observed in any group of treatments. This result also supported the proposed *in vitro* findings, indicating the high histocompatibility of PMs@NaF-SAP treatment.

Thus, PMs@NaF-SAP seems to perform better than CHX because the oral cavity is a mixture of complex microbial communities. Topical PMs@NaF-SAP treatment can efficiently suppress the incidence and development of dental caries without disrupting the ecological balance of the oral microbiota or having deleterious effects on surrounding soft tissues *in vivo*. These *in vivo* results agree with the *in vitro* data, demonstrating that this material could be a suitable next-generation anti-caries material and promote restoration in dentistry.

## 4 Conclusion

This study develops a novel SAP-decorated bacteria-responsive on-demand multidrug delivery system for the prevention of dental caries and promotion of enamel restoration. The system uses cariogenic acidic pH as a trigger and MAL-modified PEG-*b*-PLL/PBA-sheddable micelles as nanocarriers loaded with the antibacterial drug TA and the restorative drug NaF. Additionally, bio-inspired SAP DpSpSEEKC is connected to the micellar nanoparticles for the specific adhesion to tooth. Attributed to the strong enamel adhesion of the peptide and the pH-cleavable boronate ester between TA and PBA, PMs@NaF-SAP can stick to the tooth surface and tolerate the buffered effect of saliva in the oral environment, accelerate the release of TA and NaF onto caries occurring along with the oral microenvironment acidizing. Both *in vitro* and *in vivo* measurements have confirmed the intelligent drug-released on demand system exerts effective antibacterial adhesion and cariogenic biofilm resistance, inhibits enamel demineralization and promotes remineralization to prevent tooth decay and promote enamel restoration. Cytotoxicity tests, microbiome and histological analyses showed few adverse effects of the nanosystem on cells, oral microbiota diversity, and gingival and palate tissues.

Compared to current antibiotics or restorative approaches, the system has many advantages in terms of specific identification of cariogenic conditions, intelligent on-demand drug release, and resistance against salivary buffering while maintaining oral microbiota diversity. Therefore, the topical use of PMs@NaF-SAP can be introduced to expand the few clinically available options for caries prevention and defect restoration therapy. Application to rodent caries models facilitates the development of clinically effective mouthwash, spray, paint or related anti-caries products. This system is expected not only to provide guidance and support for the innovation of the existing post-defect restoration strategies but also to constitute major progress of construction and clinical transformation of smart controlled drug delivery in diseases prevention and restoration.

## Conflict of interest

The authors declare that they have no known competing financial interests or personal relationships that could have appeared to influence the work reported in this paper.

## Ethics approval and consent to participate

All experiments received ethics approval and consent to participate. Animal experiments were permitted by the School and Hospital of Stomatology, Wuhan University.

## CRediT authorship contribution statement

**Yue Xu:** Conceptualization, Investigation, Methodology, Project administration, Software, Validation, Writing – original draft. **Yuan You:** Methodology, Investigation, Visualization, Software. **Luyao Yi:** Formal analysis, Data curation. **Xiaoyi Wu:** Software. **Yaning Zhao:** Visualization. **Jian Yu:** Methodology. **He Liu:** Writing – review & editing. **Ya Shen:** Writing – review & editing. **Jingmei Guo:** Supervision, Investigation, Project administration, Writing – review & editing. **Cui Huang:** Conceptualization, Funding acquisition, Supervision, Project administration.

## Declaration of competing interest

The authors declare that they have no known competing financial interests or personal relationships that could have appeared to influence the work reported in this paper.
