# Supplementary material for: Dental plaque-inspired versatile nanosystem for caries prevention and tooth restoration
Source: Bioact Mater. 2022 Jun 21;20:418–33. doi: 10.1016/j.bioactmat.2022.06.010 (PMC9233191; doi:10.1016/j.bioactmat.2022.06.010)
Supplement: Multimedia component 1 [file mmc1.docx]

## Supplementary Information

**Dental plaque-inspired versatile nanosystem for caries prevention and tooth restoration**

Yue Xu,^1^ Yuan You,^1^ Luyao Yi,^1^ Xiaoyi Wu,^1^ Yaning Zhao,^1^ Jian Yu,^1^ He Liu,^2^ Ya Shen,^2^ Jingmei Guo ,^1^*and Cui Huang ^1^*

^1^ The State Key Laboratory Breeding Base of Basic Science of Stomatology (Hubei-MOST) & Key Laboratory for Oral Biomedical Ministry of Education, School & Hospital of Stomatology, Wuhan University, Wuhan, China.

^2^ Division of Endodontics, Faculty of Dentistry, the University of British Columbia, Canada.

*Corresponding Authors

*E-mail addresses:* guojingmei@whu.edu.cn (J. Guo), huangcui@whu.edu.cn (C. Huang)

**
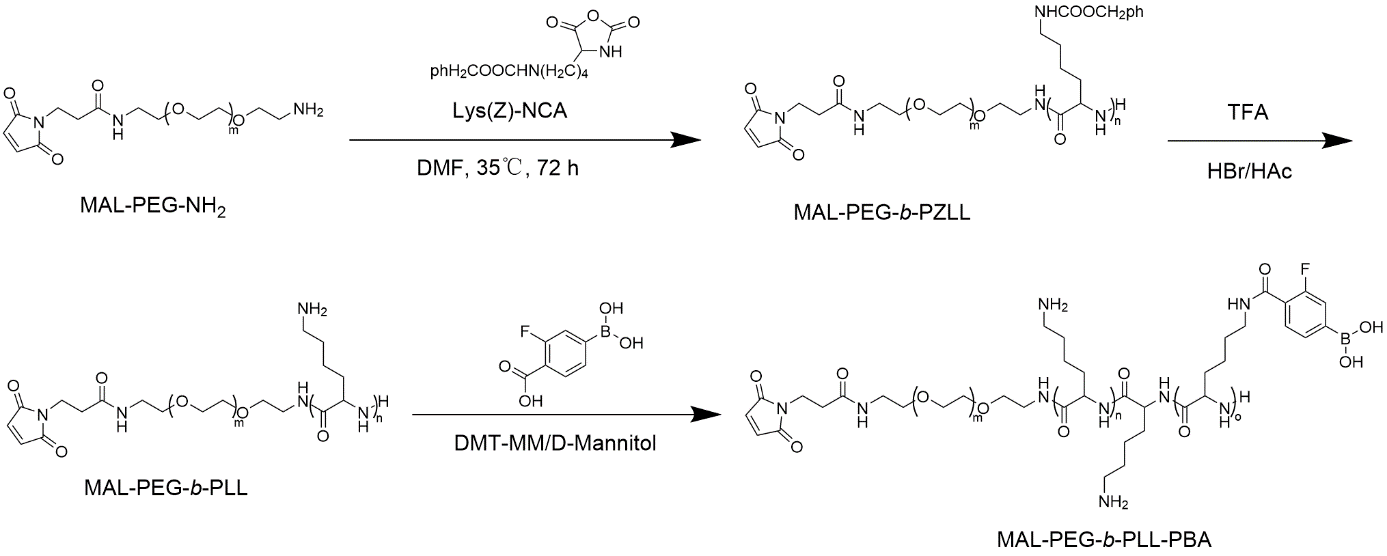
**

**Fig. S1.** Synthesis of MAL-PEG-*b*-PZLL, MAL-PEG-*b*-PLL and MAL-PEG-*b*-PLL-PBA.

**
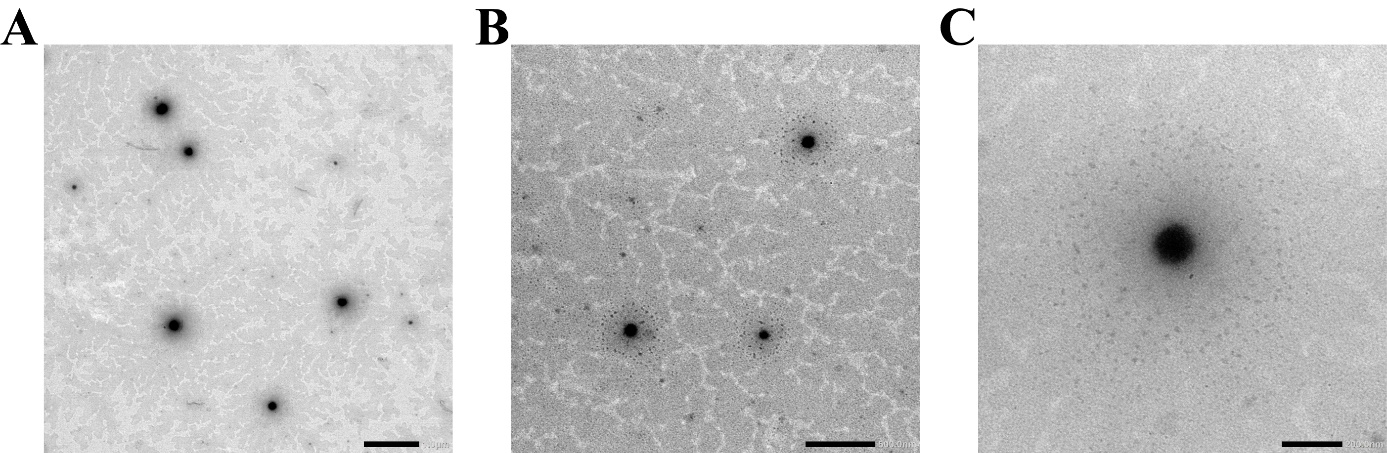
**

**Fig. S2.** The TEM images of MAL-PEG-*b*-PLL/PBA (scale bar for A: 1 μm, B: 500 nm, C: 200 nm).

**
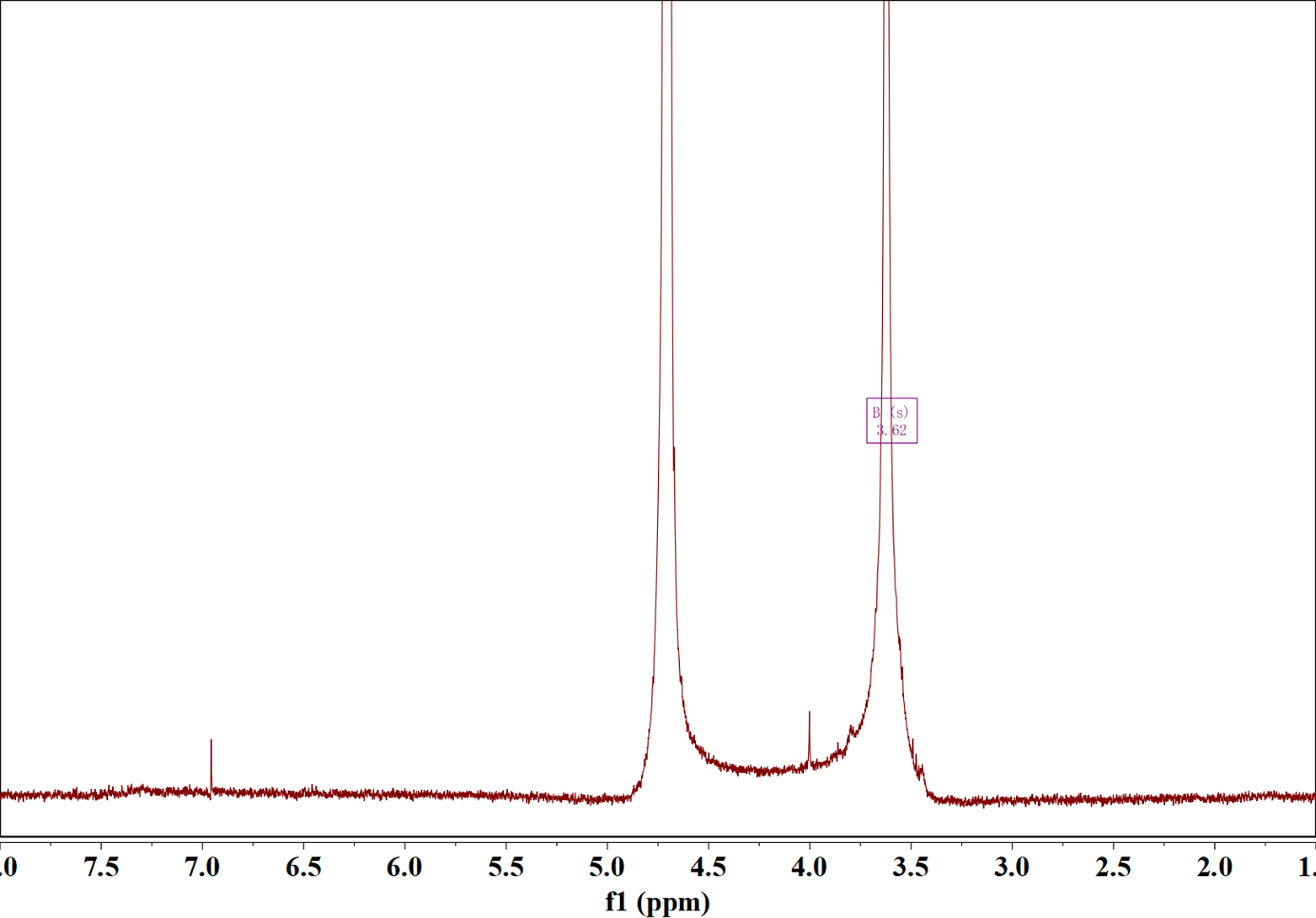
**

**Fig. S3.** ^1^H NMR spectra of PMs.

**
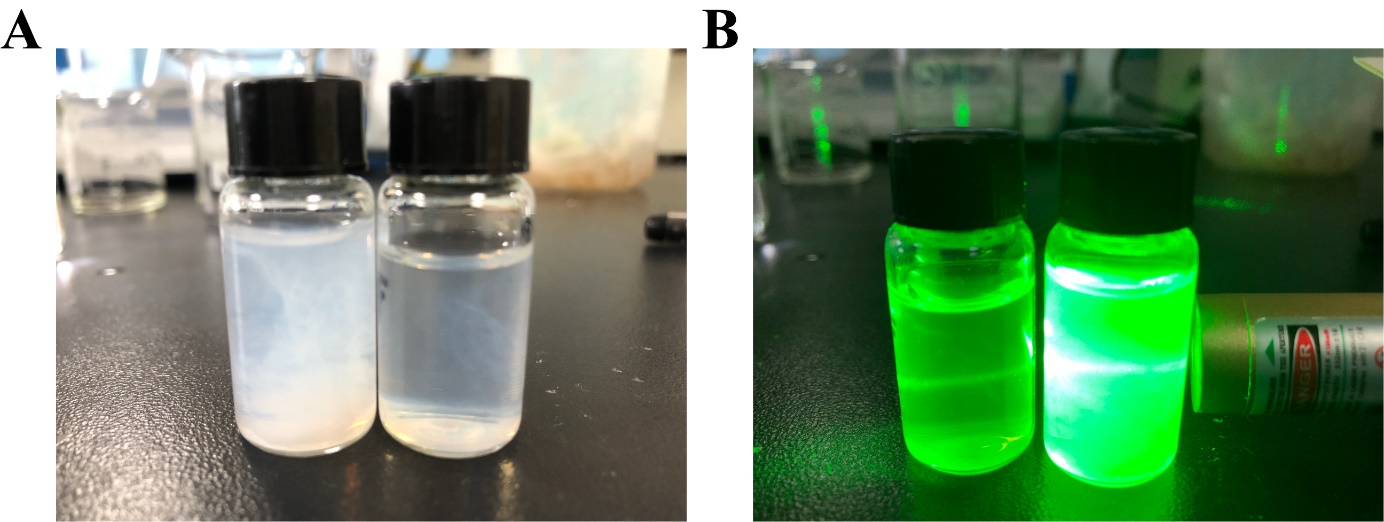
**

**Fig. S4.** Representative images of PMs@NaF nanoparticles upon synthesis. A) Photos of the micelles formation by mixing MAL-PEG-*b*-PLL/PBA and TA in NaF aqueous solution, showing the solution changed from transparent to oyster white. B) Photos of the micelles irradiated by laser, which showed the Tyndall effect that examining the successful formation of micelles.

**
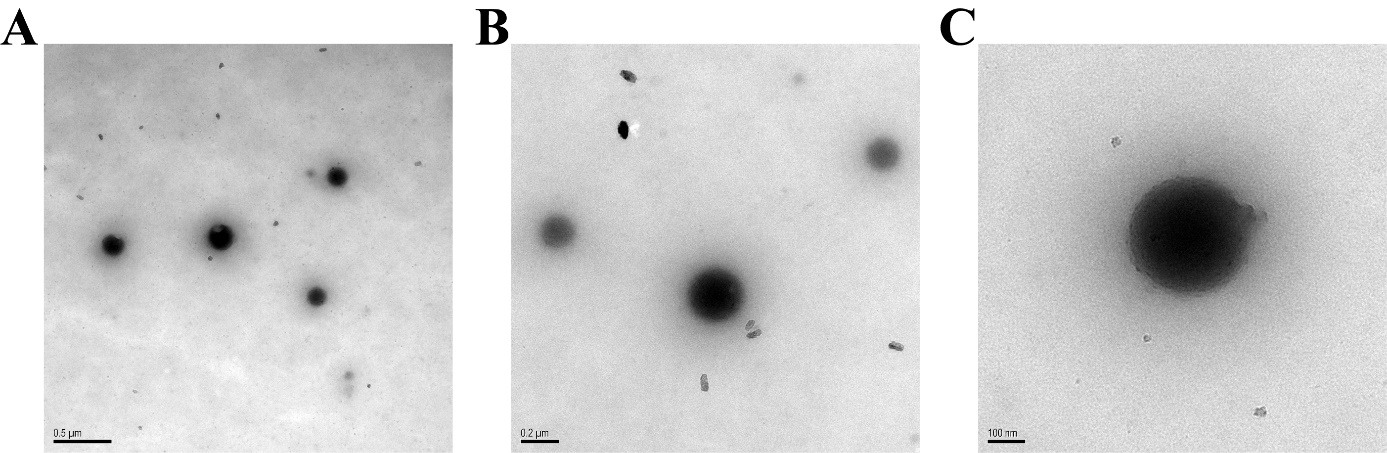
**

**Fig. S5.** The TEM images of PMs@NaF (scale bar for A: 0.5 μm, B: 0.2 μm, C: 100 nm).

**

**

**Fig. S6.** FT-IR spectra of MAL-PEG-*b*-PLL/PBA, PMs and PMs@NaF.

**Fig. S7.** Drug loading of PMs@NaF (unconjugated with SAP) *in vitro*.

**Fig. S8.** Cumulative *in vitro* release of TA from PMs@NaF at different pH values.

**Fig. S9.** Cumulative *in vitro* release of NaF from PMs@NaF at different pH values.

**
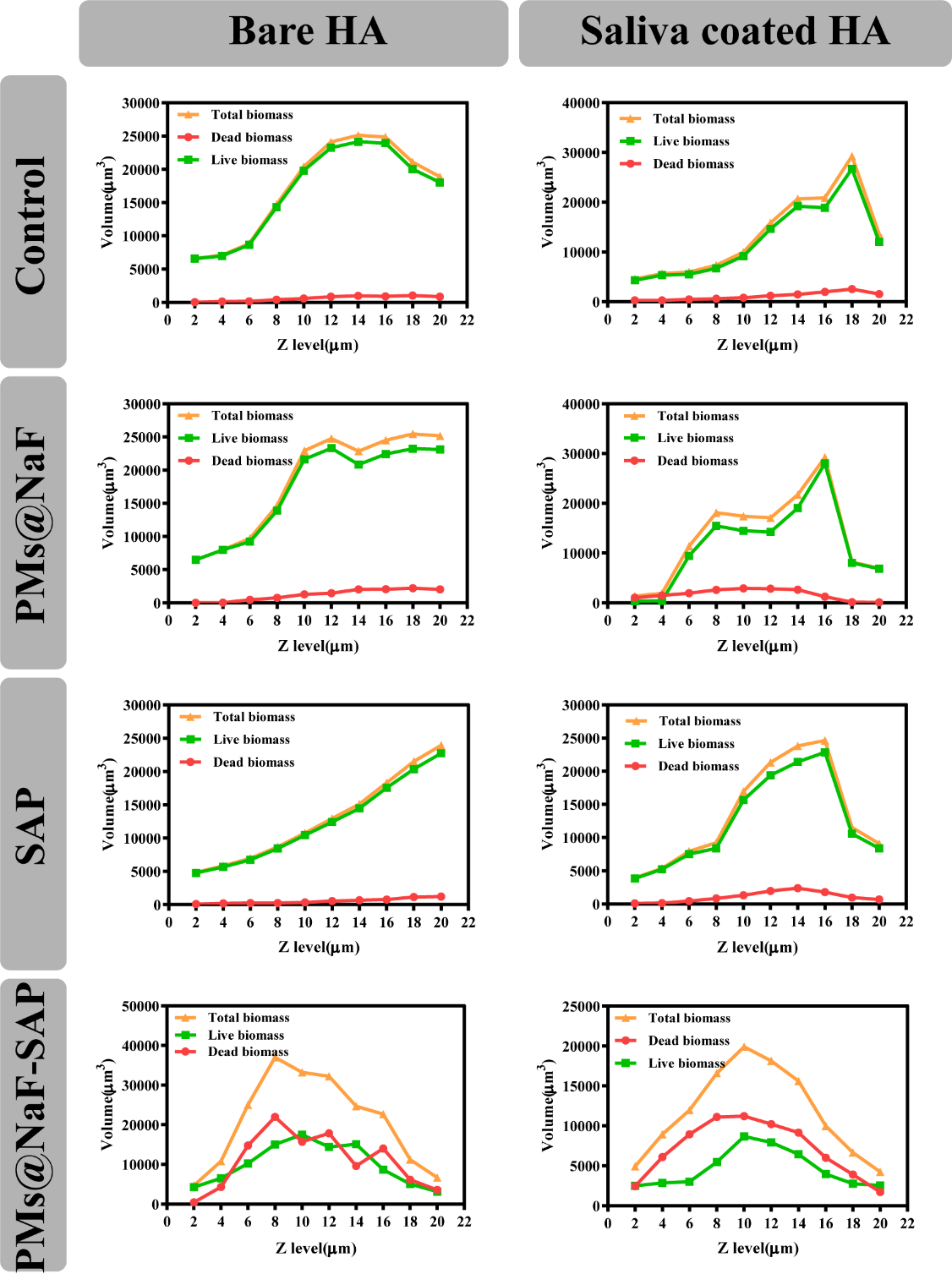
**

**Fig. S10.** Adhesion of *S. mutans* on HA and sHA discs evaluated by fluorescence intensity analysis after pre-treated with sterile PBS (control), PMs@NaF, SAP or PMs@NaF-SAP solutions.

**
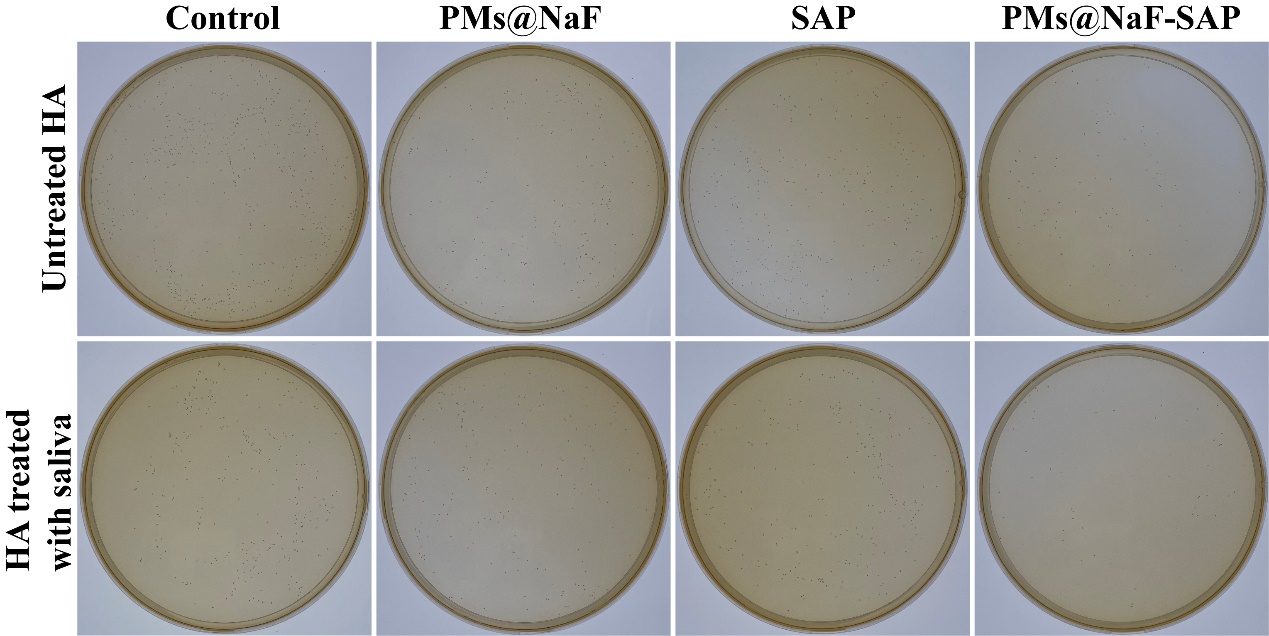
**

**Fig. S11.** Photographs of *S. mutans* colonies grown on BHI agar plates in antibacterial adhesion assay.

**
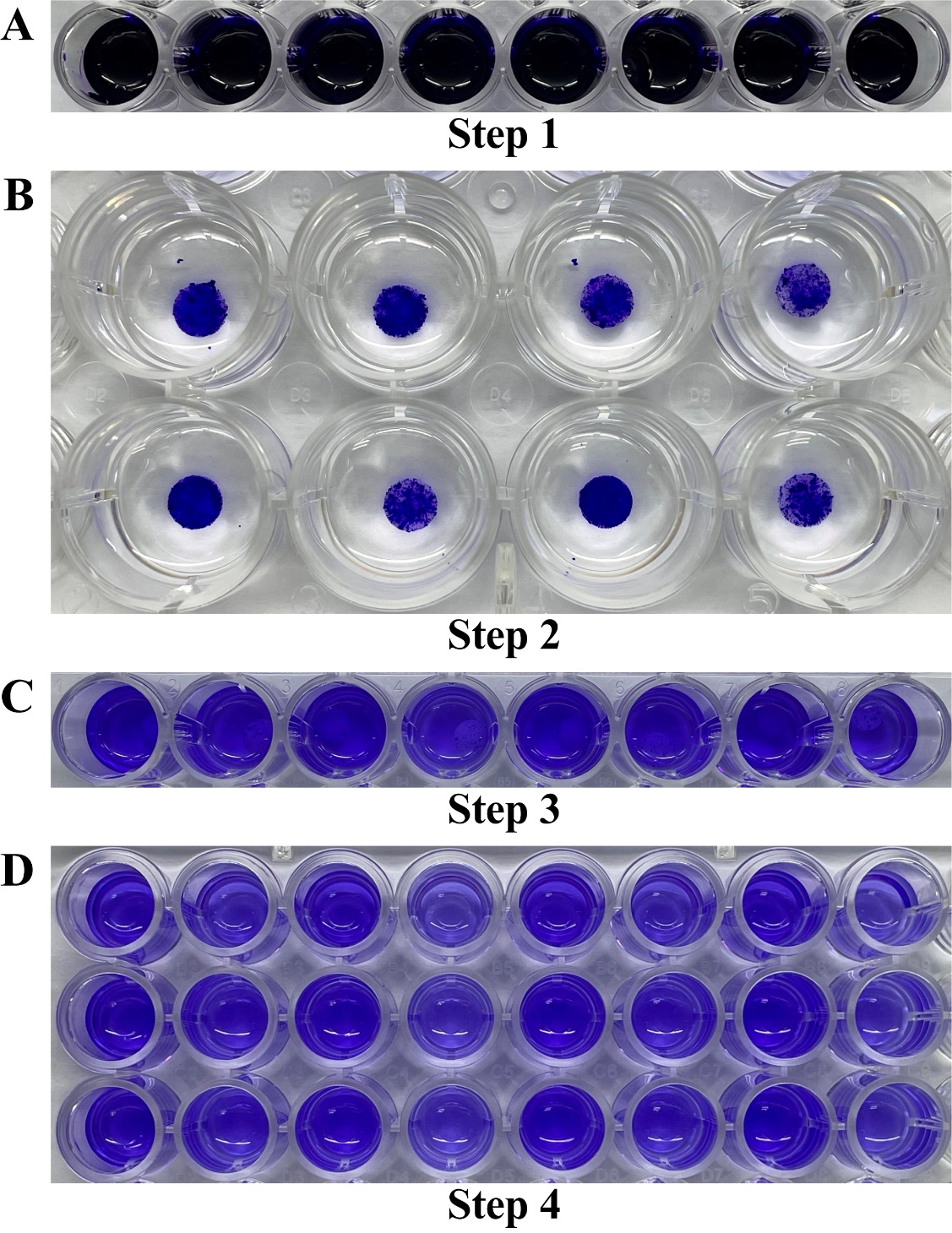
**

**Fig. S12.** Photographs of crystal violet staining steps in antibacterial adhesion assay.

**
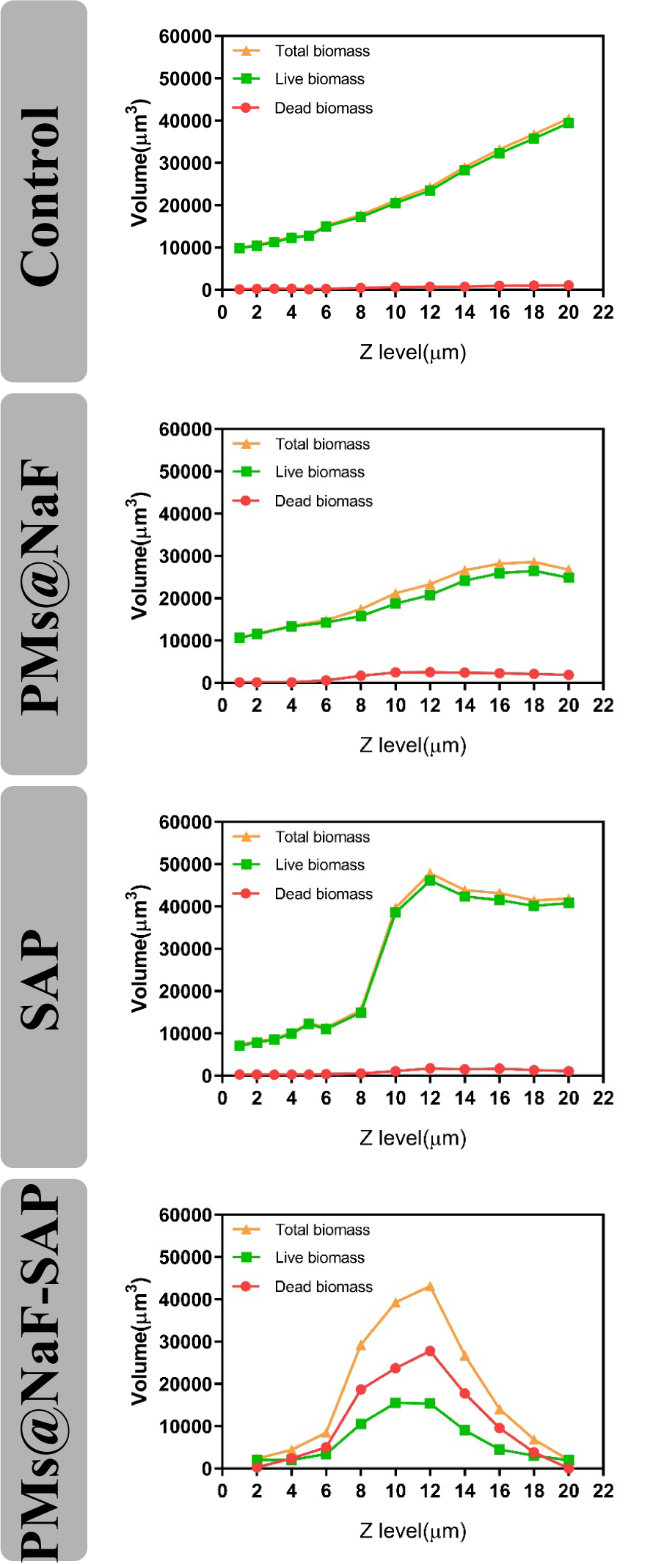
**

**Fig. S13.** The penetration and antibiofilm activity of sterile PBS (control), PMs@NaF, SAP or PMs@NaF-SAP solution treating on the pre-established *S. mutans* biofilm evaluated by fluorescence intensity analysis.

**
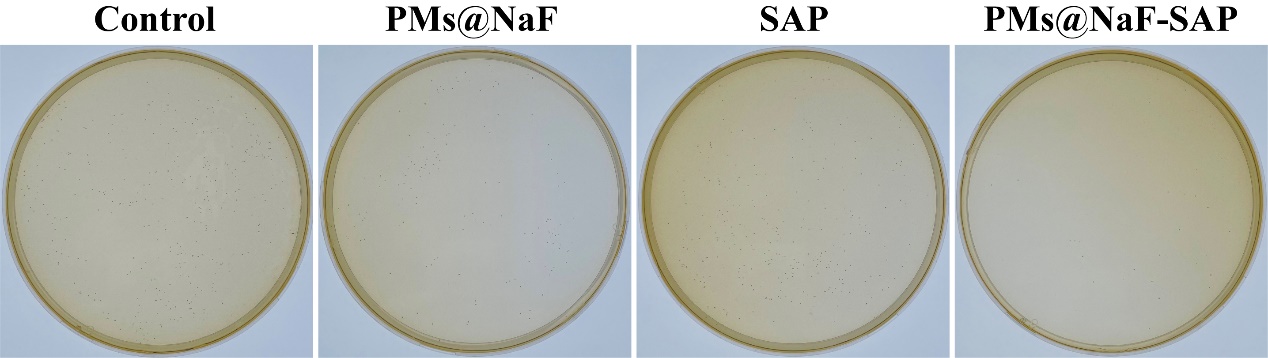
**

**Fig. S14.** Photographs of *S. mutans* colonies grown on BHI agar plates in cariogenic resistance assay.


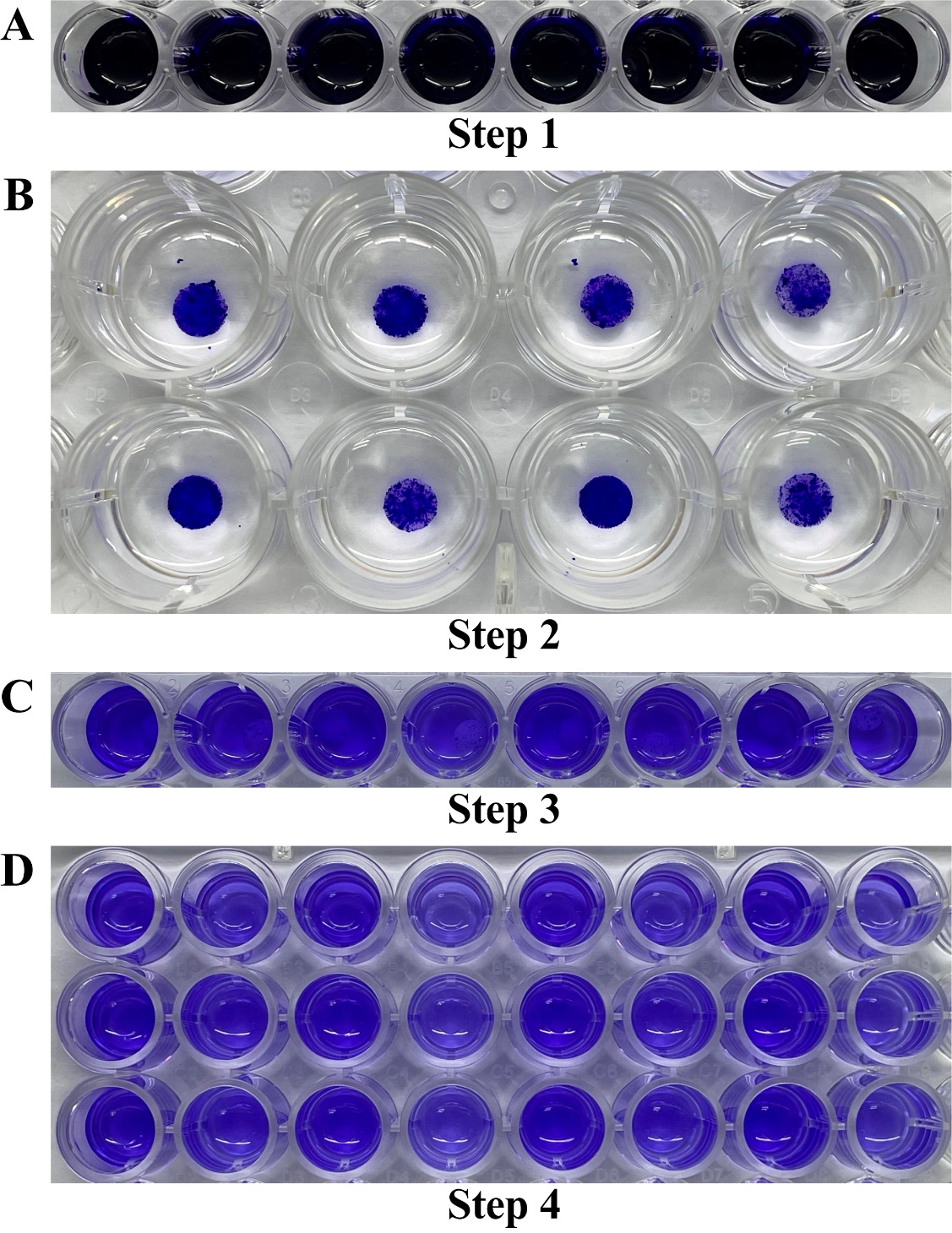


**Fig. S15.** Photographs of crystal violet staining steps in cariogenic resistance assay.

**
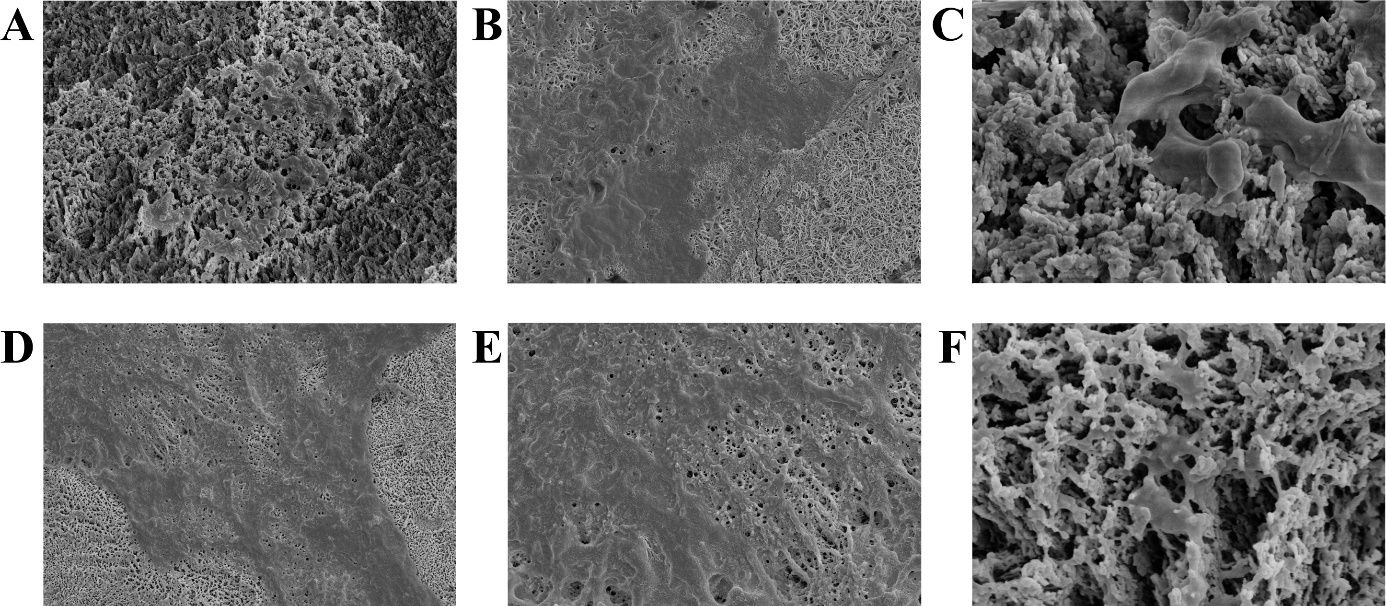
**

**Fig. S16.** SEM of SAP group (A-C) and PMs@NaF-SAP group (D-F) showed materials adhering to the enamel.

**
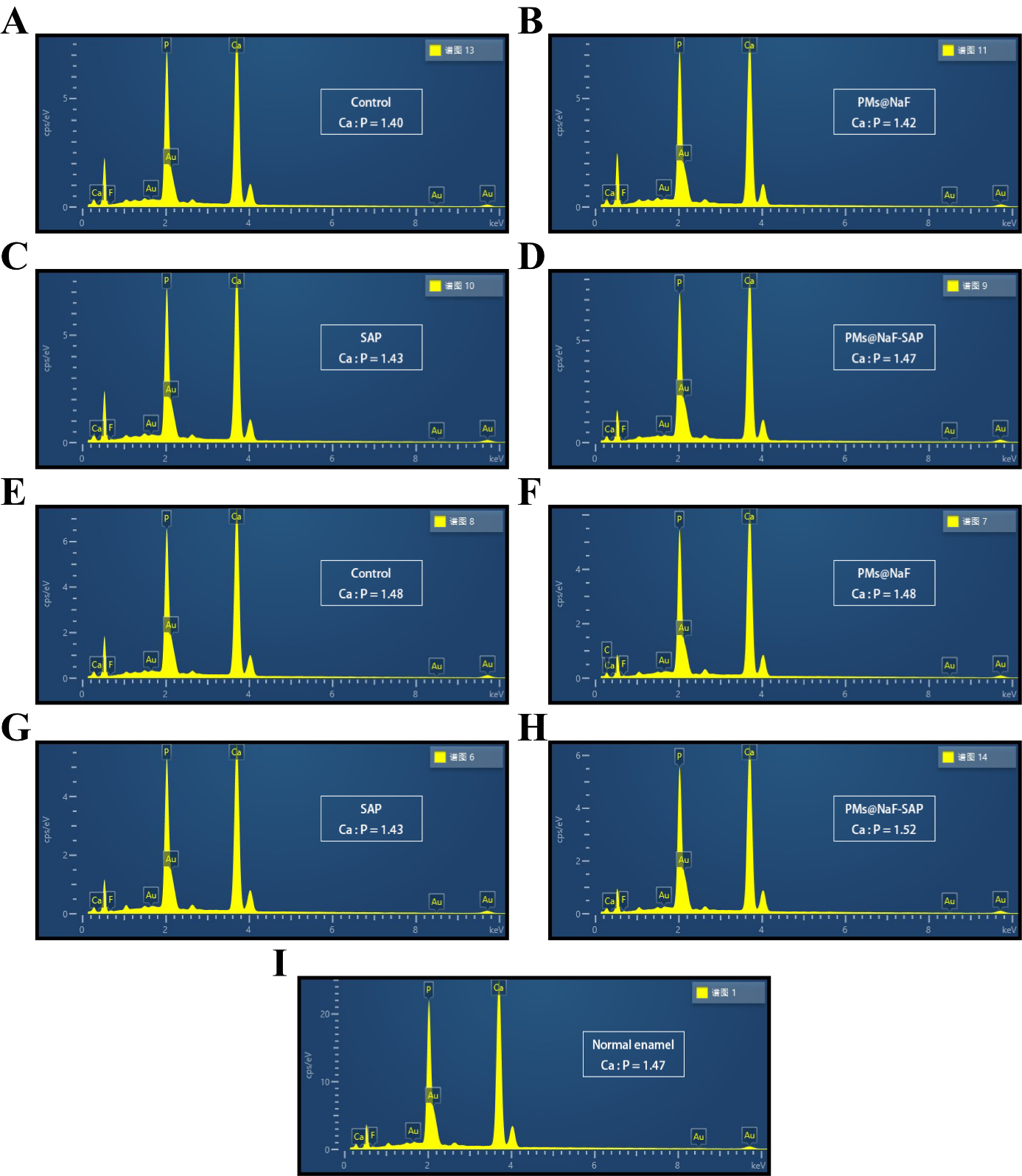
**

**Fig. S17.** EDS of different groups in demineralization (A-D) and remineralization (E-H) assay, in comparation with the normal enamel (I), indicating the Ca/P ratio of enamel surface after demineralization or remineralization treatment.

**
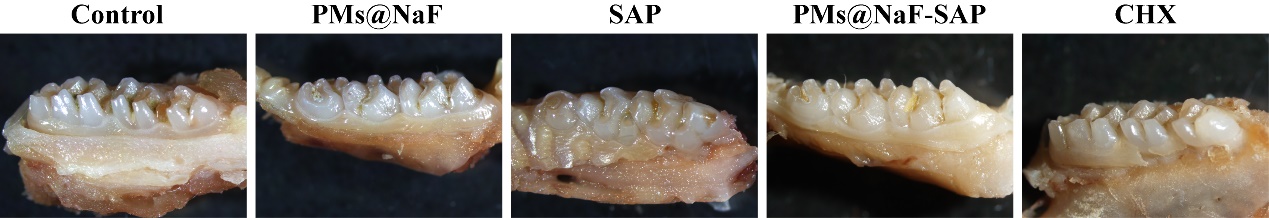
**

**Fig. S18.** Representative images of molars taken by stereomicroscope.

**
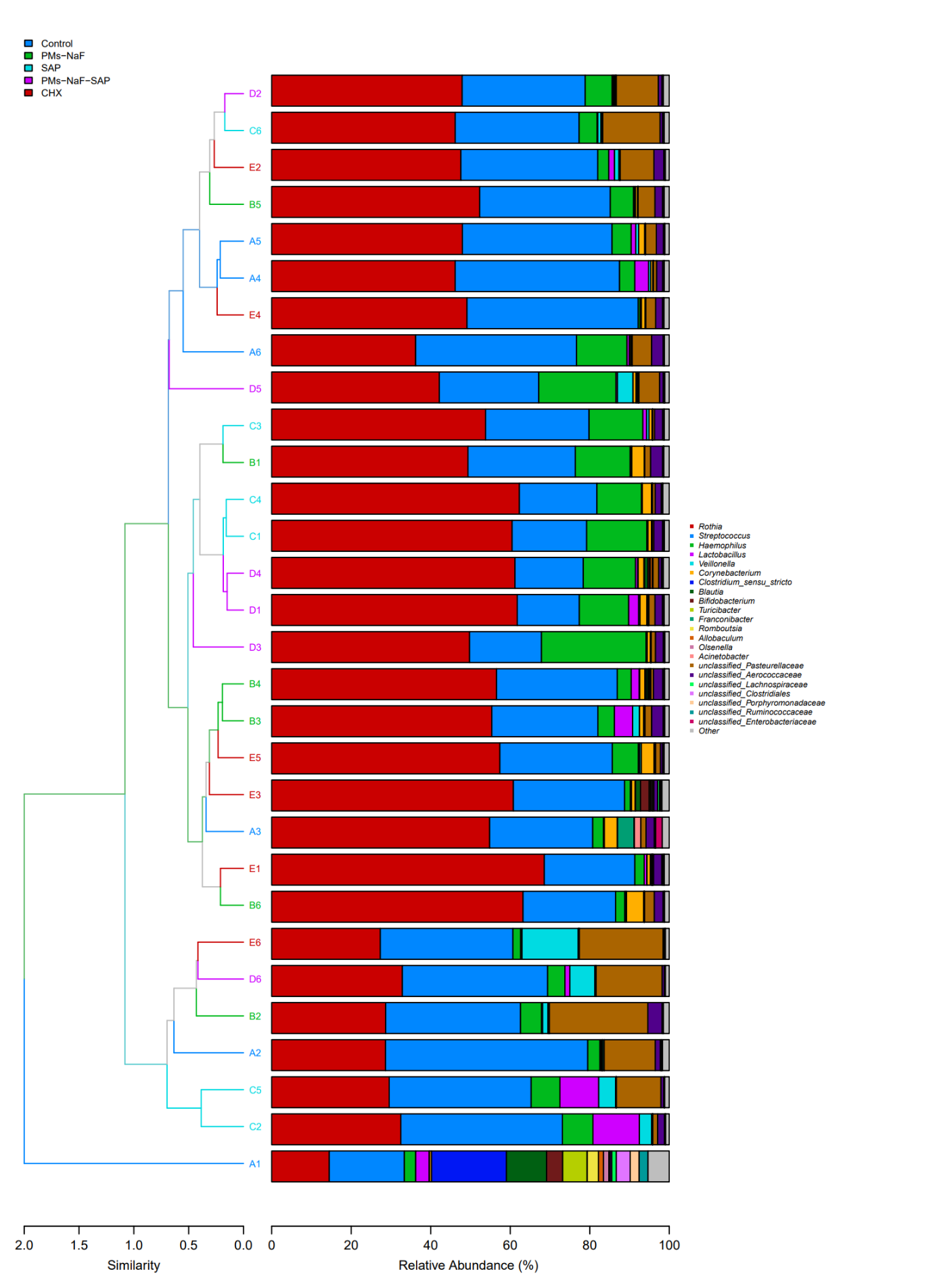
**

**Fig. S19.** Combined analysis of sample clustering tree and histogram.

**
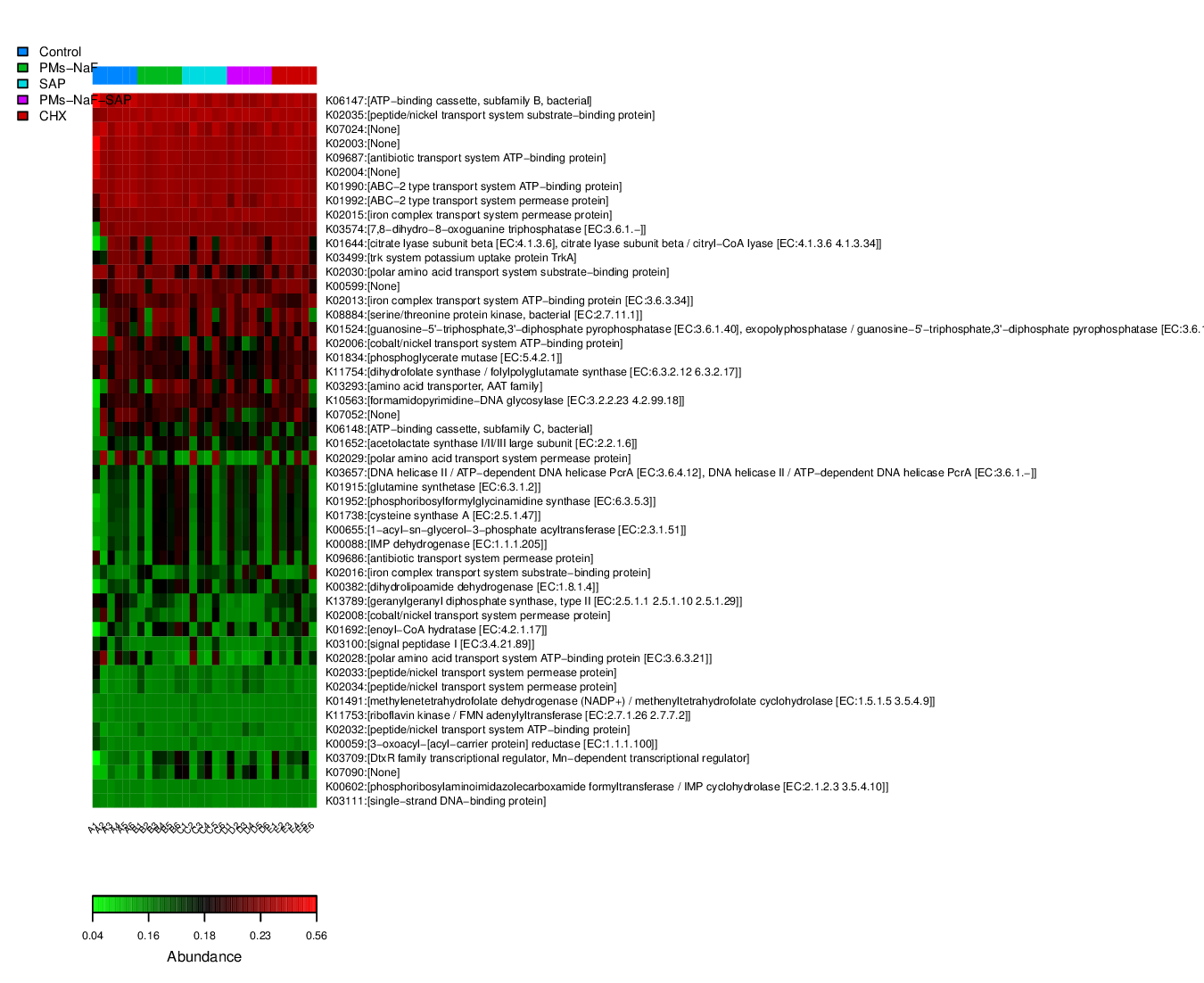
**

**Fig. S20.** Functional abundance heatmap.

**Table S1:** Nucleotide sequences of primers that were used in PCR test.

| Gene | Primer sequences | Amplicon (bp) |
| --- | --- | --- |
| 16SrRNA  gtfB  gtfC  comD  comE  luxS | F: 5’-CCATGTGTAGCGGTGAAATGC-3’  R: 5’-TCATCGTTTACGGCGTGGAC-3’  F: 5’-AGCCGAAAGTTGGTATCGTCC-3’  R: 5’-TGACGCTGTGTTTCTTGGCTC-3’  F: 5’-TTCCGTCCCTTATTGATGACATG-3’  R: 5’-AATTGAAGCGGACTGGTTGCT-3’  F: 5’-TTCCTGCAAACTCGATCATATAGG-3’  R: 5’-TGCCAGTTCTGACTTGTTTAGGC-3’  F: 5’-TTCCTCTGATTGACCATTCTTCTG-3’  R: 5’-GAGTTTATGCCCCTCACTTTTCAG-3’  F: 5’-CCAGGGACATCTTTCCATGAGAT-3’  R: 5’-ACGGGATGATTGACTGTTCCC-3’ | 144  123  122  113  147  148 |
